# Fusing appearance and vein morphology using dual-branch deep networks for accurate medicinal plant identification

**DOI:** 10.3389/frai.2026.1771431

**Published:** 2026-04-17

**Authors:** Chembon Rajeendran Karthik, Parthiban Maheswari Adithya, Naveen Nidadavolu, Ananthakrishnan Balasundaram, Ayesha Shaik

**Affiliations:** 1 School of Computer Science and Engineering, Vellore Institute of Technology (VIT), Chennai, Tamil Nadu, India; 2 Centre for Cyber Physical Systems, Vellore Institute of Technology (VIT), Chennai, Tamil Nadu, India

**Keywords:** deep learning, DenseNet, dual branch network, leaf vein pattern, MobileNet

## Abstract

Accurate identification of medicinal plants from leaf images is essential for pharmacognosy, biodiversity conservation, and agricultural decisions. But, accurate identification of medicinal leaves still poses a potential challenge in real-world conditions due to high similarity between species, variability within classes, uneven lighting, background clutter, partial views and occlusions. Existing RGB-based deep models often overfit to color-texture cues that vary with environmental conditions, whereas venation-based (skeleton) methods provide anatomically stable morphology but inherently suppress the critical appearance information needed to distinguish visually similar species. In this study, we introduced a novel dual-branch deep learning framework that explicitly separates and preserves appearance and venation learning using two independent pre-trained feature extractors, instead of relying on traditional fusion methods that combine the modalities at the input level or compress both cues into a single fused image stream. Specifically, MobileNetV2 is used to capture global appearance descriptors (texture, pigmentation, and shape), while DenseNet121 learns fine-grained vascular topology from skeletonized vein representations; the resulting embeddings are then combined via late feature-level fusion to form a unified discriminative representation that minimizes modality interference and maximizes complementarity. To further improve robustness and reduce bias introduced by dataset imbalance, we have integrated a class-frequency aware augmentation strategy that adaptively strengthens minority-class transformations while preserving majority-class fidelity, alongside transfer learning, class weighting, and regularization. The proposed approach is trained and evaluated on a curated dataset of 14,344 paired RGB-skeleton images spanning seven medicinal plant species. It is rigorously benchmarked against RGB-only, skeleton-only, and fused image baselines. Experimental results have shown that the proposed dual-branch model achieves 97 % overall accuracy with high precision, recall, and F1-score, showcasing that the structured dual-stream learning of appearance and vein morphology provides a solution for medicinal plant recognition with the potential for robust performance in changing and real-world settings.

## 1 Introduction

In traditional healthcare systems worldwide, medicinal plants play a vital role. Despite advances in modern medicine, an estimated 80% of people in various regions of Asia and Africa still receive their primary medical care from traditional medicine and herbal therapies (WHO, 2020). About 70% of individuals in rural India alone use plant-based remedies for routine medical needs (Kumar and Chandel, 2018). Rural India has approximately 70% of its population using plant-based treatments for their primary health care needs (Kumar and Chandel, 2018). As the economy grows, forecasts predict that the herbal medicine market which was USD 70.6 billion in 2023 is projected to grow to USD 328.7 billion by 2030 (Global Herbal Market Report, 2023), and the higher classification of medicinal and aromatic plants market, the entire market, is expected to be approximately USD 967 billion by 2035 (Herbal Industry Forecast, 2024). These numbers illustrate not only the cultural significance of the medicinal plant resources but also the commercial significance. However, traditional methods of plant identification, based on taxonomic keys and expert morphological assessment, are often slow, labor-intensive, and subject to human error. Furthermore, manual classification becomes even more difficult for medicinal plants due to high intra-class similarity (different species share similar leaf shapes) and inter-class variability caused by environmental factors such as disease, nutrient availability, and seasonal changes (Atique et al., 2018; Tan and Chang, 2019). For instance, depending on their maturity and growing circumstances, leaves from the same medicinal species might vary in color (green, yellow, or brown) and texture, and distinct species may have similar surface appearances, making correct identification more difficult (Pushpa and Rani, 2021). By directly learning discriminative features from leaf photos, recent advances in deep learning and computer vision have enabled automatic plant classification with high accuracy. To extract color, texture, and shape data from RGB photos, early methods have shown promise in classifying plant species (Kadir et al., 2015). However, performance in real-world situations may suffer due to RGB-based models' sensitivity to occlusion, background clutter, and uneven illumination (Zhang et al., 2017). However, as a strong alternative modality, leaf venation patterns—which are frequently species—specific and persistent in a range of lighting conditions—have been investigated (Atique et al., 2018; Tang et al., 2023). Nonetheless, a framework based on venation may overlook essential visual cues, such as color and texture, that can distinguish morphologically indistinct species (Tan and Chang, 2019). A single modality of visual information will not suffice to account for the full suite of discriminative cues necessary for reliable classification of medicinal plants, whether by traditional RGB or by venation alone. This has led to interest in two-branch deep learning paradigms that can incorporate the complementary benefits of skeletal leaf venation and RGB images, thus reducing some incompleteness issues of single-modality methods while increasing robustness in classification processes and contexts beyond a given environmental scene. The objectives of this study are to develop a two-branch deep learning model to classify the medicinal plant specimen dataset by RGB and skeletal leaf images, and to use transfer learning to collect features from state-of-the-art pre-trained convolutional backbones for each modality. Furthermore, to improve classification accuracy, the study intends to develop a feature fusion method to concatenate RGB and venation-based features and to compare the proposed two-branch, two-feature fusion with single-branch baselines on a custom medicinal plant dataset. To classify medicinal plant species, we propose a robust dual-branch deep learning architecture that takes RGB leaf images and skeletonized vein structures as inputs. We have developed a model that overcomes the limitations of a single-modality approach, including cigarette sensitivity, changes in lighting in the RGB data, and the loss of texture in vein reconstruction of the leaf description. In this manner, both the snapshot of color–texture properties and the stable morphological vein pattern provide complementary information to improve model predictions. The technique combines lightweight, high-performance convolutional backbones with transfer learning, enabling it to be used in resource-constrained contexts. The proposed framework provides a dependable and scalable solution for medicinal plant identification in applications ranging from herbal pharmacognosy to biodiversity conservation and agricultural monitoring, as demonstrated by experimental evaluation on a curated dataset of 14,344 paired RGB–skeleton images. The proposed study provides the following contributions:

1. A dual-branch deep learning approach that facilitates multi-class classification of medicinal plant leaves by integrating RGB appearance information from RGB images with 3D skeletonized vascular (or venation) structures, allowing improved classification accuracy of species with high morphological similarity and/or significant visual variability.
2. A strategy for building a comprehensive, diverse dataset that results from combining multiple public datasets of medicinal plants to create paired RGB image and skeletal image datasets using a standardized preprocessing and vein extraction pipeline, thus allowing for a consistent representation of features across all classes.
3. A feature-level fusion framework that uses the complementary capabilities of MobileNetV2 and DenseNet121 to provide global and structural descriptors, thus increasing Model Robustness against variations in luminosity, noise, partial occlusions, and naturally occurring environmental variation.
4. A thorough comparative evaluation of the performance of the proposed dual-branch architecture against the performance of strong single-modality baselines. Results show that the proposed approach has the potential to improve class-wise stability across visually and morphologically similar species under variable image conditions, even though its overall accuracy is slightly lower than that of the RGB-only baseline. These results highlight the approach as a strong complementary option for situations where detailed venation cues are essential.

## 2 Related work

In the early stages of succulent classification, researchers relied on patterns of leaf venation (Davis and Hsu, 1959; Krupanidhi et al., 2018), which are species-specific and resilient to environmental change. Atique et al. (2018) studied leaf classification by outlines of vein patterns with deep convolutional networks—ResNet and DenseNet—on the MalayaKew dataset of 44 species of tropical trees. They used Canny edge detection to isolate venation features and examined the impact of different optimizers on the classification results for vectorized leaf images. They observed a best accuracy of 95.72% from DenseNet-169 and the Adam optimizer, vs. ResNet-101's best accuracy of 89.50%, and also notable accuracy from DenseNet using the SGD optimizer at 94.20%. The researchers concluded that DenseNet outperformed ResNet and could accurately distinguish subtle differences in vascular structure. Overall, their results supported the use of vein-based features in a traditional classification approach and confirmed DenseNet's ability to discriminate plant morphology.

Tan and Chang (2019) introduced the D-Leaf framework for object-based classification of certain plant species using a series of leaf-vein measurements. In this case, they incorporated features derived from methods that used Sobel-segmented vein patterns, which encode vein-based structural components relevant to species classification. Specifically, they engaged three convolutional neural networks (CNNs), including: transferring learning from AlexNet, fine-tuning AlexNet to their own neural network application, and their own model, which they labeled D-Leaf. They classified the features acquired using the networks referred to above using Support Vector Machine (SVM), Artificial Neural Network (ANN), K-Nearest Neighbors (KNN), Naïve Bayes (NB), and CNN classifiers. In their experiments, Tan and Chang preprocessed the image data by resizing them to 250 × 250 pixels to support consistent feature extraction across images. In their results, Tan and Chang reported a testing accuracy of 94.88% for D-Leaf, which was comparable to the tuned uses of AlexNet, demonstrating that a CNN morphometric approach for the classification of plant species was effective.

Additionally, hybrid shape descriptors based on shape contours and method-based morphometric outcomes have offered a valuable approach, as evidenced by the development of the Improved Multiscale Triangle Descriptor (IMTD) (Wu et al., 2023), particularly given the shape's multi-scale features. Combining IMTD with convolutional features was found to support classification somewhat compared to using standard single-channel data derived from an RGB image, but at the cost of full loss of multi-scale properties. Furthermore, researchers (Huynh et al., 2020) published a simple but effective method for integrating vein data at the input level. They showed that, with the red channel replaced by a vein image obtained using an image analysis scheme to differentiate the veins and sent as RGB data to the CNN pipeline, performance improved dramatically on the Flavia and Swedish Leaf data sets, reaching over 98.22% accuracy. This suggested that vein structure as input for leaf morphology can help support the use of the standard CNN pipelines.

A key sub-task of conducting subsequent research is the segmentation of leaf venation. Liu et al. (2020) proposed a new segmentation algorithm using DeepLabV3+ and the MobileNetV2 backbone. They overcame several problems, such as the slow segmenting speed of leaves, partial occlusion, and measurement error. The pipeline of Liu et al. includes an algorithm, Convex Hull-Scan, to repair missing segments of repaired vein outlines, and the F-3MS refinement with Floodfill, MorphologyEx, Median blur, and Skeletonization to remove burrs from skeletal lines. The Liu et al. model achieved an MIoU of 81.5% and a mean pixel accuracy of 92.9% while segmenting leaves at 9.8 frames per second and reducing the number of parameters by 89.4%–5.8M. Measurements of leaf veins with the models showed leaf length and width accuracies of 96.36% and 96.14%, respectively.

For example, researchers (Krupanidhi et al., 2022) studied the venation patterns of ap medicinal and aromatic plants to evaluate their potential as taxonomy markers. Their study provides comprehensive descriptions of vein morphology, including primary, secondary, and tertiary venation types for each group. Because of the reliability with which individual vein patterns can be associated with species, the authors emphasized their utility for identifying plants when flowers and fruits are not available. This method is a simple, inexpensive, non-destructive initial classification approach in the fields of pharmacognosy and biodiversity conservation.

Xu et al. (2020) applied deep learning to automatically segment leaf venation networks. This study demonstrated that network-based methods can capture different vascular patterns. They leveraged the strengths of convolutional neural networks (CNN), which enhance image feature extraction in both segmentation and detection tasks. The authors approached their work by using vein regions rather than the entire veins, as had been done in previous vein studies. As a limitation, these approaches require large datasets with carefully labeled images for supervised learning. While they invite the use of high-quality manual annotation for specific patches in an image, this limits their utility for scaling up as a general methodology in their case.

Ensemble and multimodal fusion methodologies have achieved some of the highest classification accuracies, as exemplified by the ensemble deep learning-automated leaf medicinal identification framework developed by Sachar et al. (2021). In this study, a set of three convolutional neural network (CNN) architectures (MobileNetV2, InceptionV3, and ResNet50) is combined with weighted average techniques to facilitate better overall classification while applying transfer learning. This study acquired feature data from leaf images of 30 medicinal plant species, and classification was performed using a SoftMax layer after a dense layer extracted features. This approach of applying an ensemble methodology was verified using 3-fold and 5-fold cross-validation, with an impressive test accuracy of 99.66%, and every test instance for the ensemble method outperformed any of the individual models. This work convincingly demonstrates the ability of ensemble learning to combine the strengths of multiple CNN architectures.

Researchers (Lapkovskis et al., 2021) developed a multimodal deep learning framework that integrates separate modalities of the plant—a flower, leaves, fruit, and stem—into a single identification task. Their proposed deep learning framework uses supervised learning with MobileNetV3 backbones to extract features from different plant organs, which are then fused into a multimodal representation. By leveraging variability in plant organs, the framework is more robust to occlusions, seasonal changes, and interspecific variation. The authors experimentally showed that the multimodal fusion model outperformed when using single plant organ features and demonstrated that different visual cues can improve plant identification.

The literature review on dual-branch fusion research has recently been updated to include Bi et al. (2022) and their proposed DualCMNet, a lightweight dual-branch pipeline for recognizing maize varieties. Using both hyperspectral and color data, the two branches have a 1D-CNN for the hyperspectral branch and MobileNet-V3 for the color spatial features branch. To accomplish this, the model needs a set of new modules; namely, HShuffleBlock, which ensures that the feature dimensions align, and the channel-spatial attention (CBAM) mechanism and gated fusion unit or other attention mechanisms, such as input feature similarity, but for the fusion of information in the attention mechanism, in which region features are weighted for relevance. Their model has only 2.53 million parameters but achieves high performance (98.75%) using 5-fold cross-validation and outperforms single-modal networks. The authors determine that the compact size of the models is a promising capability for agronomic Internet of Things applications for deployment at the edge.

Similarly, researchers (Tang et al., 2023) introduced MPR-net, a dual-branch attention fusion network for recognizing medicinal plants. The goal of MPR-net is to achieve a balance between accuracy and efficiency. In addition to MPR-net, the authors prepared the Karst Landform Herbs Dataset, a large benchmark comprising 120 plant species and 56,650 multi-organ images captured in their natural habitats. MPR-net achieves this balance of accuracy and efficiency by leveraging local feature extraction via convolutional approaches and global token-based modeling. MPR-net integrates attention fusion-based methods, such as Window multi-head self-attention and pooling-based vision transformers, into its architecture. This architecture achieved a Top-1 accuracy of 94.7%, outperforming both ResNet-152 (89.2%) and ViT-Base (91.4%). It also benefits from only 21.6 million parameters and remains extremely efficient on complex background images typical of nature.

Recent developments demonstrate a growing need for lightweight, deployable applications and models. In this regard, Pushpa and Rani designed a compact DCNN entitled Ayur-PlantNet, specifically for Ayurvedic species of Indian origin that maintains a significant level of accuracy (92.27%) and remains computationally efficient (Pushpa and Rani, 2021). In another study, Schuler et al. proposed a color-aware two-branch DCNN that separates luminance and chrominance (L vs. AB channel). Schuler et al. concluded that they can achieve computational efficiency and improve robustness by using separate architectural channels, suggesting that data-type-based channels are advantageous. In an application using the PlantVillage dataset, they achieved an accuracy of 99.48%, lower than the screenshot shown for the more challenging PlantDoc dataset (Schuler et al., 2022).

Several investigations have evaluated multi-scale or hybrid venation workflows. Researchers (Karnik et al., 2022) proposed a venation-aware workflow that employs contrast enhancement, Frangi filtering, and edge extraction. They exploited multi-scale networks and attention-based dual streams that fused RGB, venation, and edge maps. The authors observed improved performance compared with traditional CNN models while keeping the model lightweight enough for mobile applications. Overall, they emphasized the effectiveness of modeling venation at multiple scales combined with texture and appearance cues. Although significant progress has been made in classifying medicinal plant leaves, various methods have been used across different studies. These range from extracting traditional morphological features to implementing advanced deep learning strategies, such as convolutional neural networks (CNNs), ensemble methods, and dual-branch fusion strategies. While all methods achieved high accuracy in a controlled lab environment, several variables still limit their application in real-world scenarios. A majority of studies relied on comparatively smaller datasets and on images obtained under controlled lighting and background conditions (Sachar and Kumar, 2022; Sachar et al., 2021; Pushpa and Rani, 2021). This limits the models' ability to generalize to complex scenarios typical in the real world, where changes in lighting, leaf orientation, occlusion, and environmental noise are common variables. Furthermore, whilst some models are considered highly accurate, they still require substantial computational power, as with ensemble-based architectures (Sachar et al., 2021) or multi-attention architectures (Attention-Based Fusion Study, 2020). This makes these models impractical for the dependent sensors on several monitoring devices, which require less computational power and are often used in field applications.

Furthermore, intra-class variance and inter-class similarity in medicinal plant leaves pose additional concerns. Seasonal shifts, adverse symptoms of disease, as well as abiotic stressors, can all impact the color and texture of the leaf (Atique et al., 2018; Liu et al., 2020; Krupanidhi et al., 2022) and were included in venation-based studies. Venation skeletons do offer greater morphological stability, but extracting them requires pre-processing steps and are prone to precision variation when image quality is uncertain, as illustrated in segmentation studies (Liu et al., 2020; Bi et al., 2022). Moreover, there is a lack of large-scale, publicly available benchmark datasets for medicinal plants, which limits the use of consistent methods for comparison or evaluation. Across the literature, studies often use small or self-compiled datasets limited to their own applications, even when evaluated in multimodal and bi-branch models that utilize a fused learning process (Lapkovskis et al., 2021; Multi-modal Fusion Study, 2021). The fusion process introduces additional redundancy or imbalance if the modes are improperly integrated, leading to overfitting or reduced reliability when applied to unobserved data. Recent research has shown that combining morphological vein properties with color and texture information can be used to classify medicinal plants. However, the field continues to face challenges surrounding classification dataset diversity, responsiveness to natural environmental changes, computational efficiency, and multimodal feature fusion. These concerns point to a need for a robust dual-branch model that leverages complementary RGB and skeletonized-vein features to classify medicinal plants and applies to real-world settings.

To address these gaps, the propose a dual-branch medicinal plant classification framework with novelties and contributions such as incorporation of an input specific dual-branch design with late stage feature fusion for complementary feature capture, a class-frequency-aware augmentation strategy for the mitigation of class imbalance in the dataset and enhancement of the recognition of rare plant species, and improved robustness to illumination variations, color changes, and partial occlusions for reliable real world performance. Ultimately, the proposed model achieved superior performance over the single-input baseline models, including strong results for morphologically similar and visually challenging plant species.

In recent years, transformer-based and hybrid deep learning approaches have also been explored for plant leaf analysis. Vision Transformer models have demonstrated improved robustness to illumination changes and background variations, particularly in agricultural disease detection tasks (Murugavalli and Gopi, 2025). Hybrid CNN–Transformer architectures have similarly shown promise by combining local texture descriptors with global structural relationships, enabling stronger fine-grained discrimination in leaf datasets (Aboelenin et al., 2025). Lightweight CNN–Transformer hybrids designed for mobile agricultural applications have also emerged, emphasizing computational efficiency and field-level deployability (Jia et al., 2025). Furthermore, transformer-driven classifiers tailored to botanical leaf images have demonstrated the growing adoption of ViT-based methods for plant recognition (Karaoǧlan and Karadeniz, 2024). These recent studies highlight a shift toward transformer and hybrid architectures in plant classification research and serve as relevant context for evaluating the design choices adopted in the present work.

## 3 Materials and methods

### 3.1 Dataset description

All experiments used a dataset comprising 14,344 RGB images and their corresponding skeletonized leaf-vein images. The dataset was divided into training, testing, and validation sets using an 80-10-10 split. This resulted in 11,475 images for training, 1,435 images for testing, and 1,435 images for validation. The training set was used to optimize the model, the validation set for hyperparameter tuning and early stopping, and the test set for the final performance evaluation. The split was stratified by class to maintain proportional representation of each plant species across subsets, and care was taken to ensure that no images from the same physical leaf instance appeared in multiple subsets, thereby preventing data leakage.

### 3.2 Dataset preparation

**Figure 1:**
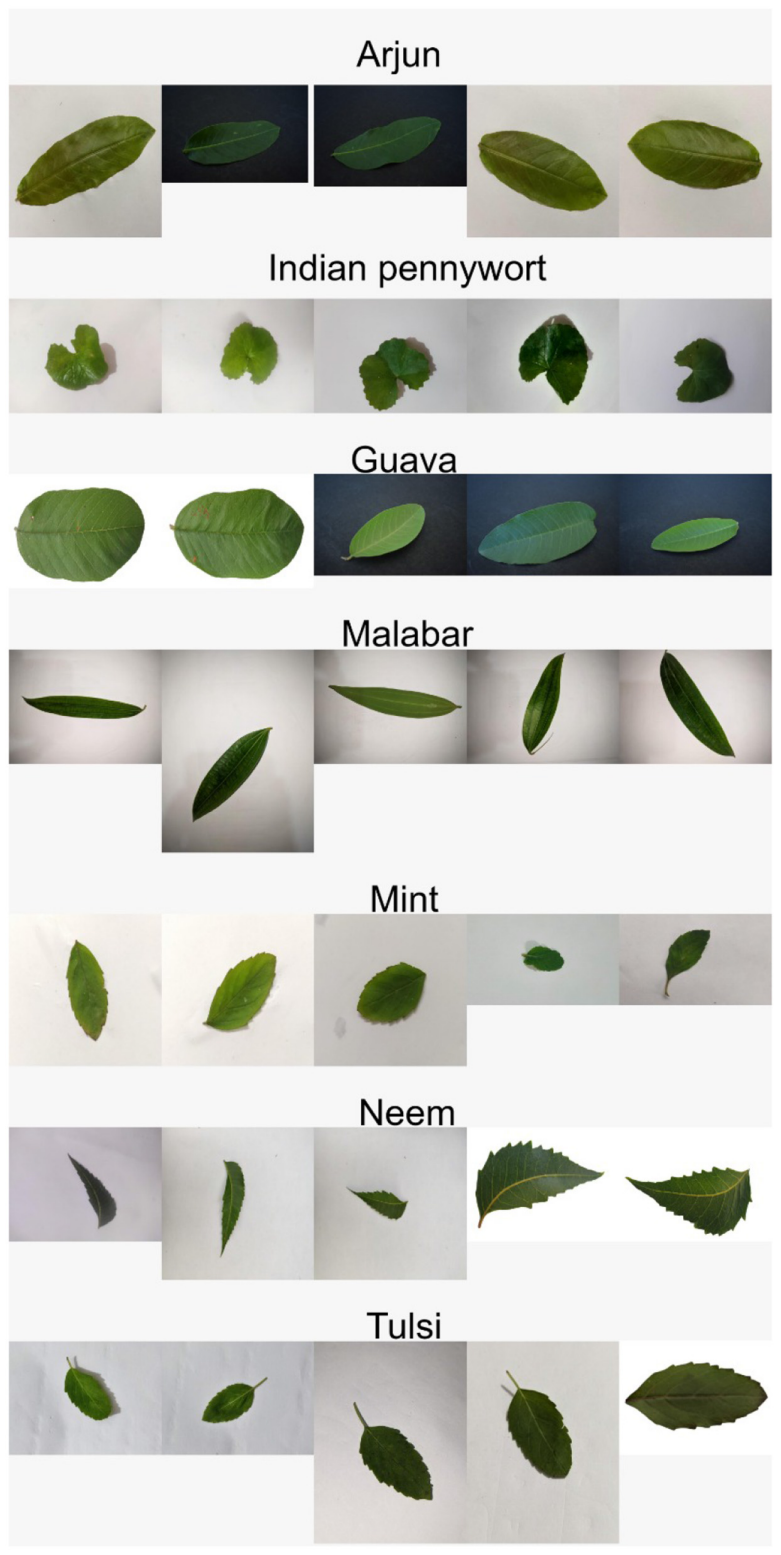
Dataset images of chosen classes.

The dataset proposed for the training of the model is shown in Figure 1. The chosen dataset is an ensemble of multiple datasets, which were obtained from Mendeley data and consist of medicinal plant leaf images of the following classes: Arjun, Guava, Mint, Malabar, Tulsi, Neem, and *Centella asiatica*. In total, about 7,172 images were collected, further investigated, and processed to extract features for training the proposed models. The datasets used are given below:

- Medicinal Plant Identification Dataset (Goyal et al., 2021): This dataset comprises labeled images of six medicinal plant classes: Arjun, Curry Marsh Pennywort, Mint, Neem, and Rubble. It comprises 9,660 images generated from 1,380 field-collected original images, augmented with standard transformations (shift, flip, zoom, shear, brightness, and rotation).
- MED_117 Medicinal plant leaf Dataset (Sarma et al., 2020): It contains leaf images of 117 medicinal plants native to Assam, India. This dataset includes approximately 77,700 raw images extracted from 10 to 15 s video recordings captured with a Canon SLR camera against a white background. Furthermore, it includes U-Net segmented images of the same.
- *Centella asiatica* Leaf image Dataset (Singh A. et al., 2020): It is a focused dataset consisting of a single plant species (*Centella asiatica*) and consists of 9,094 high-res images suitable for plant leaf area analysis, plant identification, and recognition of medicinal plants.
- Pudina leaf dataset:freshness analysis (Rana et al., 2021): It is a specialized dataset consisting of a single mint species, which analyzes the freshness condition of mint leaves across three distinct categories- dried, fresh, and spoiled states. This dataset is highly useful for quality assessment tasks, freshness detection systems, and other related applications.
- Comprehensive Herb species dataset (Ahmad, 2024): It consists of high-resolution images of five herb species-Neem, *Centella asiatica*, Coriander, Basil, and Bay leaf. Initially composed of 2,500 field-collected images, the dataset was expanded with augmentation techniques.
- A Database of Leaf images (Bholowalia and Kumar, 2017): Consists of 4,503 professionally captured leaf images of 12 species of plants and divides images into two categories: healthy and diseased. The images were captured using a Nikon D5300 camera under controlled conditions. This dataset supports plant species identification and also disease diagnosis research.
- Medicinal Leaf dataset (Chaudhary et al., 2020): Consists of 1,500 high-quality images of leaves from 30 medicinal plant species, with about 60–100 image samples per species. The images were captured on a Samsung S9+ camera from mature leaves obtained from local gardens and standardized by slight rotations and tilts.

Many datasets were used to obtain greater variation and diversity in the leaves of the same species. The images in the proposed dataset showed considerable variations. Some were captured under strong illumination, such as Sunlight, while others were taken in Shaded conditions. The image backgrounds also varied: some were plain monochromatic, while others had leaf shadows; some contained noise, such as simple Gaussian noise, while others had none. Although such variabilities can enhance the model's adaptability, they may degrade training performance. To mitigate these issues, image processing techniques were applied to the images to remove noise, backgrounds, and shadows, thereby standardizing the dataset. This enabled us to obtain a clean, high-quality leaf image with a monochromatic background, facilitating the easier, more effective extraction of leaf features.

The split of the image data by class is listed in Table 1, including the leaf name, the number of images, and the dataset used.

**Table 1:** Image data per class.

Name of leaf	Amount of images	Datasets referenced
Guava (*Psidium guajava*)	450	A database of leaf images (Bholowalia and Kumar, 2017), Medicinal leaf dataset (Chaudhary et al., 2020)
Arjun (*Terminalia arjuna*)	347	Medicinal plant identification (Goyal et al., 2021), A database of leaf images (Bholowalia and Kumar, 2017)
Indian pennywort (*Centella asiatica*)	1,044	Comprehensive herb species (Ahmad, 2024), *Centella asiatica* leaf image (Singh A. et al., 2020)
Malabar (*Cinnamomum tamala*)	889	MED_117 Medicinal plant leaf (Sarma et al., 2020), Comprehensive herb species (Ahmad, 2024)
Neem (*Azadirachta indica*)	1,412	Comprehensive herb species (Ahmad, 2024), Medicinal leaf dataset (Chaudhary et al., 2020), Medicinal plant identification (Goyal et al., 2021)
Mint (*Mentha*)	1164	Pudina leaf dataset: freshness analysis (Rana et al., 2021), Medicinal plant identification (Goyal et al., 2021)
Tulsi (*Ocimum sanctum*)	1,866	Comprehensive herb species (Ahmad, 2024), A database of leaf images (Bholowalia and Kumar, 2017), Medicinal leaf dataset (Chaudhary et al., 2020)
Total	7,172	

From the table above (Table 1), it is evident that the Guava and Arjun classes had the fewest images, and to compensate, various augmentation techniques were applied before providing the data to the model.

**Figure 2:**
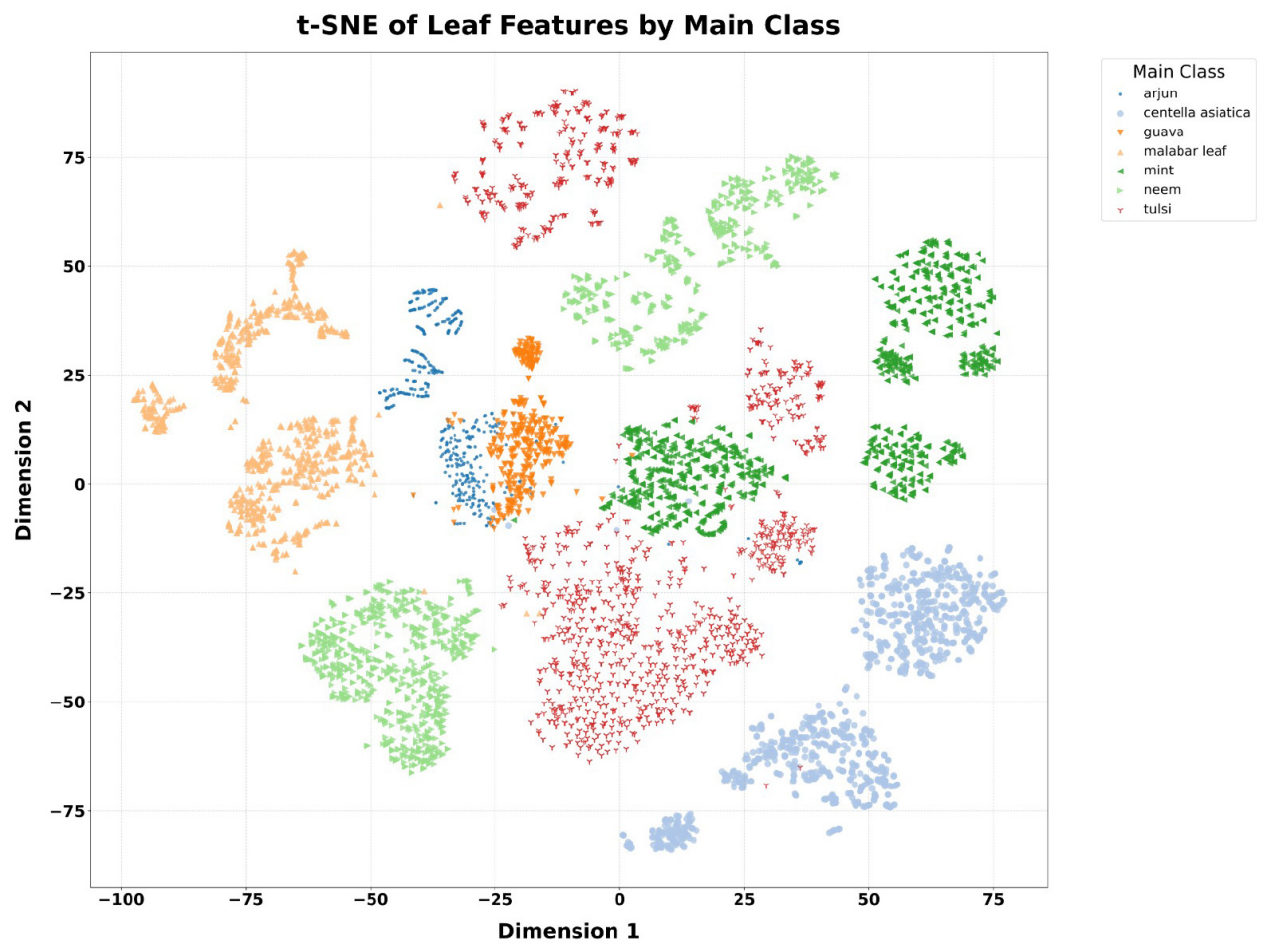
t-SNE visualization of RGB leaf feature embeddings by plant species.

To evaluate the separability of these seven medicinal plant classes in the feature space before the model's training phase, t-SNE (t-distributed Stochastic Neighbor Embedding) dimensionality reduction was applied to the high-dimensional feature embeddings extracted from the RGB images of the leaves. The reason for using t-SNE as a dimensionality reduction technique is that it can project high-dimensional feature vectors into a 2-dimensional space while preserving neighborhood relationships. The required feature vectors for the t-SNE backbone network are derived from a CNN applied to the RGB image. The CNN backbone network and its role in the overall framework are discussed in detail in Sections 3.2 and 3.3. The t-SNE visualizations are depicted in Figure 2.

Figure 2 indicates that Neem samples form two well-separated clusters with low dispersion, indicating highly consistent and distinctive RGB features across its samples. *Centella asiatica* similarly produces compact, isolated groupings spread across the lower portion of the embedding space, reflecting low intra-class variance and strong inter-class separation from other species. Guava and Malabar leaf both have relatively light groupings. However, Malabar leaf exhibits a broader spatial spread, suggesting intra-class variability likely attributable to differences in leaf maturity and capture conditions across the source datasets. Arjun appears as a loose, elongated scatter rather than a single, compact cluster, indicating notable intra-class variability within the species. Tulsi occupies a large, diffuse region in the center of the embedding space, exhibiting substantial intra-class diversity and multi-dataset origins. Mint forms several subclusters which partially overlap with the Tulsi embedding region in the center, indicating that these two species share similar RGB appearance features- this finding directly motivates that the inclusion of the skeleton vein branch in the proposed dual-branch framework, as the use of the vein morphology provides the discriminatory signal that the RGB features alone cannot reliably supply for this species pair.

Subsequently, the leaf dataset was further processed to obtain the required features and images for use as inputs to the training model. First, image processing techniques were used to extract leaf-venation features from the leaf dataset and present them as a binary leaf image comprising the leaf outline and vein structure. To obtain this, a series of filters was used to produce the final leaf-venation binary image; the steps are shown in the figure.

**Figure 3:**
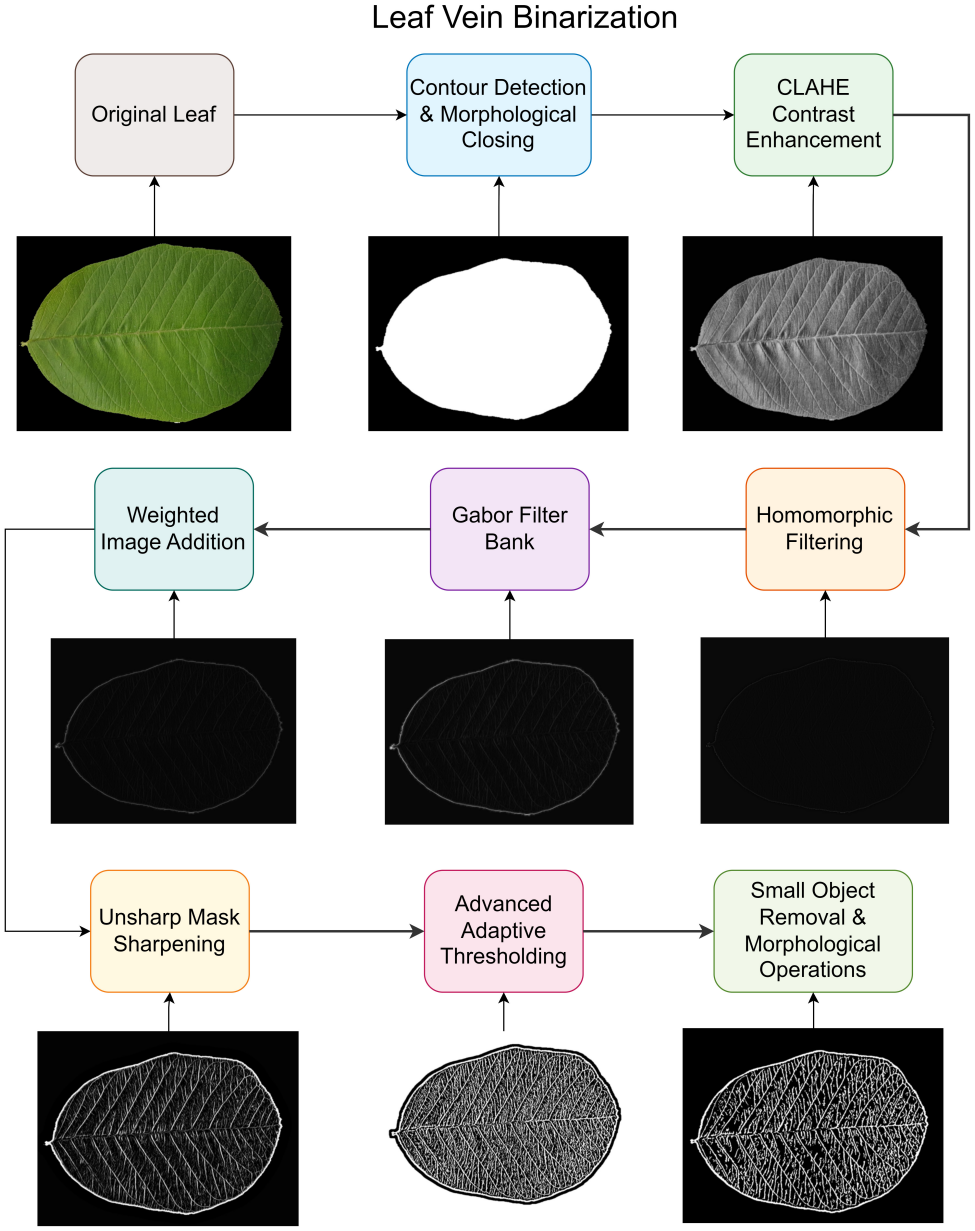
Leaf vein binarization process flow.

Each stage of the leaf vein binarization processing flow, as shown in Figure 3, is detailed as follows:

Original leaf: The processed leaf with the removed noise, background, and other abnormalities comprises the Original leaf, and this serves as the input for the vein processing pipeline. Contour Detection and Morphological Closing- Otsu's algorithm (Otsu, 1979) has been utilized, which determines the optimum threshold by maximizing the inter-class variance between foreground and background pixels. This approach searches for the threshold that minimizes the inter-class variance. The largest defined contour represents the primary leaf structure, which is then refined using morphological closing, which fills small gaps and holes within the mask using an elliptical structural element, ensuring complete leaf boundary enclosure necessary for subsequent processing steps. The formulae for the morphological closing are defined in Equation 1 as:


$$\begin{array}{l} A\cdot B=\left(A⊕B\right)⊖B \end{array}$$ (1)

where *A* represents the binary image, *B* represents the structuring element,⊕ represents dilation and ⊖ represents erosion

CLAHE (Contrast Limited Adaptive Histogram Equalization): It divides the image into contextual regions and performs histogram equalization on each region separately. Furthermore, bilinear interpolation transitions are performed between adjacent tiles.CLAHE is performed with the formulae represented in Equation 2 as:


$$\begin{array}{l} T\left(x,y\right)=\min\left(\text{CDF}\left(I\left(x,y\right)\right),\text{ClipLimit}\right) \end{array}$$ (2)

where CDF denotes the cumulative distribution function, and ClipLimit prevents excessive contrast enhancement. This prevents over-enhancement in uniform regions by clipping the histogram at a predefined limit before redistribution.

Homomorphic Filtering–Utilizes a multiplicative illumination-reflectance image model by converting it to an additive form through logarithmic transformation. The high-pass filter enhances reflectance components (leaf details) while suppressing low-frequency illumination variations. Exponential transformation recovers the enhanced image with normalized lighting conditions. This is represented with the formulae shown in Equation 3:


$$\begin{array}{l} H\left(u,v\right)=B+A\left[1-\exp\left(-k\frac{D{\left(u,v\right)}^{2}}{D_{0}^{2}}\right)\right] \end{array}$$ (3)

*A* controls the amplification of the high-frequency components, *B* regulates the low-frequency illumination components, *D*(*u, v*) represents the Euclidean distance from the frequency domain center, *D*_0_ represents the cutoff frequency of the homomorphic filter, and *k* determines the sharpness of the filter transition.

Gabor Filter Bank—the Gabor filters (Daugman, 1985) combine Gaussian windowing with sinusoidal carriers for the detection of oriented patterns. This uses multiple filters with different orientations (0, 30, 60, 90, 120, and 150) and scales to capture vein structures at various angles and widths. The maximum response preserves the strongest vein evidence while supporting background noise. This is represented by the formulae in Equation 4:


$$\begin{array}{l} g(x,y;\theta ,\lambda ,\psi ,\sigma ,\gamma )=\exp(-\frac{x^{\prime 2}+\gamma^{2}y^{\prime 2}}{2\sigma^{2}})\cos(2\pi \frac{x^{\prime }}{\lambda }+\psi ) \end{array}$$ (4)

where *x*′ = *x*cos(θ)+*y*sin(θ) and *y*′ = −*x*sin(θ)+*y*cos(θ), *f* denotes the frequency, and γ denotes the aspect ratio.

Weighted image addition—Utilizes a linear combination of the information from the homomorphic and Gabor filters (Daugman, 1985). Using an equal-weighting strategy, we can balance broad structural information with fine details of venation. Through this fusion approach, the frequency-domain enhancements and spatial-domain texture characteristics are preserved, enabling a representation suitable for subsequent processing stages. The formula in Equation 5 represents this:


$$\begin{array}{l} I_{\text{combined}}=w_{1}\cdot I_{\text{homo}}+w_{2}\cdot I_{\text{gabor}}+\gamma \end{array}$$ (5)

where *w*_1_+*w*_2_ = 1, and typically *w*_1_ = *w*_2_ = 0.5, while γ represents an optional bias term.

Unsharp Mask Sharpening—Through this subtractive enhancement technique, a high-pass filter is created by subtracting Gaussian-blurred content from the original image. Through this method, high-frequency details, such as edges and fine structures, were amplified while preserving low-frequency information. Selective application of this technique to the leaf interior prevents halo artifacts at boundaries, while the use of strength parameters controls the intensity of the enhancement. Using this technique, we were able to increase acutance without adding new detail, resulting in sharper edges through local contrast enhancement. This is shown by the formula in Equation 6:


$$\begin{array}{l} I_{\text{sharp}}=I+\alpha \left(I-I_{\text{blur}}\right) \end{array}$$ (6)

where *I*_blur_ shows the smoothed version of the original image being obtained using a low-pass filtering operation, and α controls the sharpening intensity.

Advanced Adaptive thresholding- Local thresholding adapts to varying illumination by computing pixel-specific thresholds from neighborhood statistics. Contrast stretching, achieved through percentile-based normalization, improves dynamic range before thresholding. Using this approach, we can handle uneven lighting, shadowing, and varying leaf-surface properties better than global methods, which are of paramount importance for leaf-vein detection. This is shown by the formula in Equation 7:


$$\begin{array}{l} T\left(x,y\right)=\frac{1}{N}\sum \left(i,j\right)\in N\left(x,y\right)I\left(i,j\right)-C \end{array}$$ (7)

where N(x,y) shows the local neighborhood, and N represents the size of the neighborhood, and C represents a constant offset

Small Object removal and Morphological operations: Connected component analysis is used to identify and eliminate noise artifacts below a specified area threshold, thereby preserving meaningful vein structures. The use of the morphological opening function removes the thin noise connections while maintaining the vein continuity. It is represented by the formula in Equation 8:


$$\begin{array}{l} A○B=\left(A⊖B\right)⊕B \end{array}$$ (8)

where the opening (°) consists of erosion(⊖) followed by dilation(⊕) The use of Median filtering provides final smoothing without edge blurring, which is crucial for preserving vein topology. Using this multi-step noise-reduction technique, we obtained clear binary images while preserving vein topology.

A simplified step-by-step pipeline is presented, which separates the leaf from the background and cleans it using contour detection and morphological operations. Further enhancement of the image was performed using contrast improvement (CLAHE) and lighting corrections (homomorphic filtering). The vein patterns were highlighted using multi-dimensional Gabor filters (Daugman, 1985) and fused with other enhanced outputs. Then the images were sharpened using the unsharp masking algorithm and finally converted to clean binary images using adaptive thresholding and noise removal techniques.

**Figure 4:**
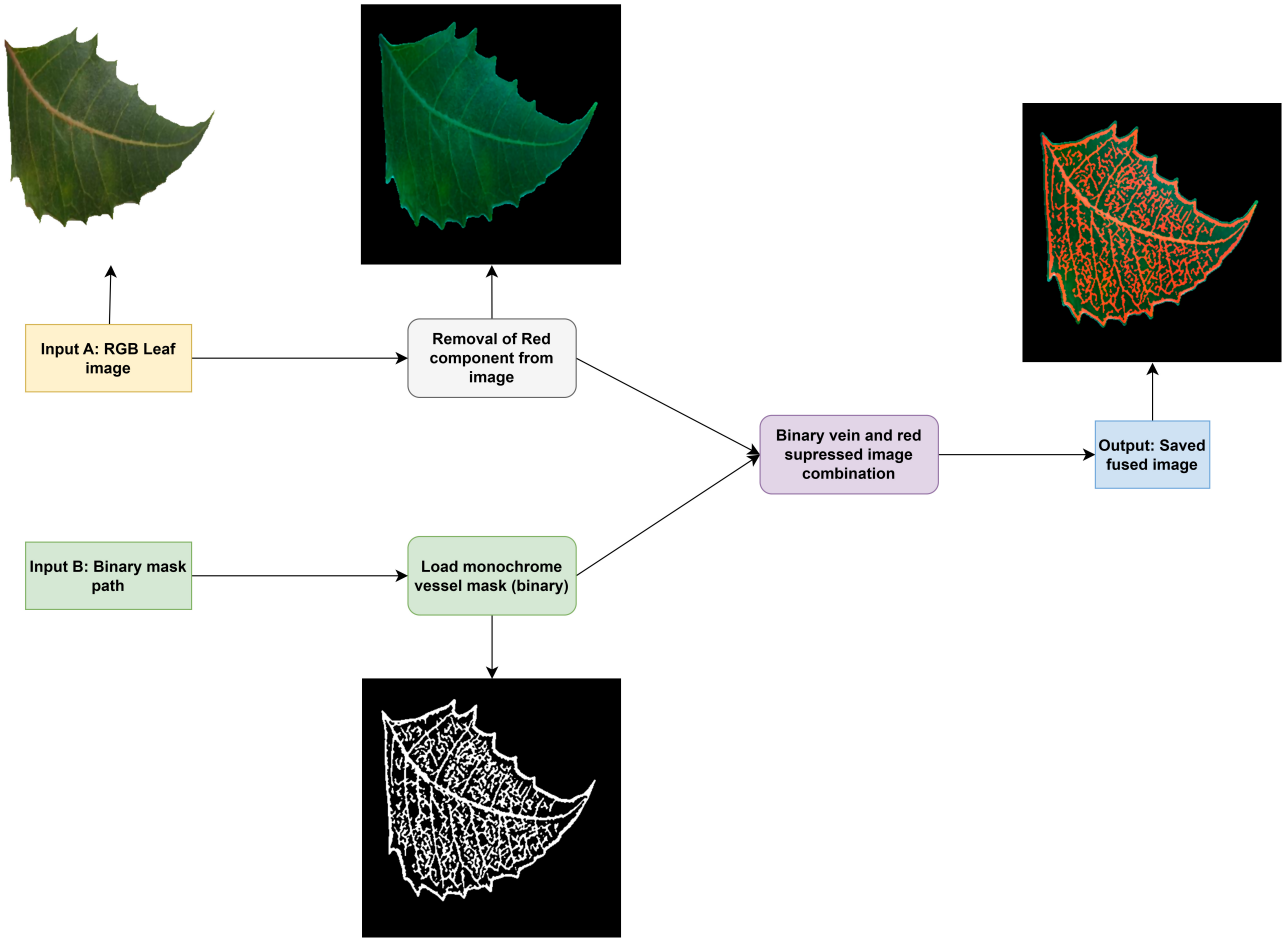
Fused leaf process flow.

After the processing stage, we obtained another set of 7,172 images, which we used for our model. Furthermore, another processing step was performed on the colored leaf image to obtain an image that consists of the original leaf image, with the red part replaced by the binary vein image we obtained above. The entire processing stage for the procurement of the fused leaf is shown in Figure 4.

The fused leaf processing consists of the stages shown in Figure 4, where two inputs are provided to the processing pipeline: an RGB leaf image and a Binary vein skeleton image. The RGB leaf image undergoes a processing step in which its red component is removed, leaving only the blue and green values. Side by side, the loaded binary leaf vein skeleton image is processed and prepared for combination, where it is cropped and fitted to match the removed red component image. To ensure that the RGB image and the extracted skeleton vein image correspond correctly, the skeleton image was generated directly from the same RGB leaf image using the same leaf mask, so both images naturally share the same leaf shape and position. Before the fusion process, both images were resized to the same resolution, and the skeleton image was cropped to the same bounding box of the leaf region obtained during segmentation. This guarantees that the vein structure is placed at the correct location within the RGB leaf, ensuring pixel-level alignment between the two inputs and preventing mismatches between the two branches.

Following this step, both images are combined in such a way that the veins are represented by the red color parts in the resulting leaf, and the resulting leaf image is the output fused leaf image. Through this process, another 7,172 images were generated and used to train models.

### 3.3 Overview of proposed work

**Figure 5:**
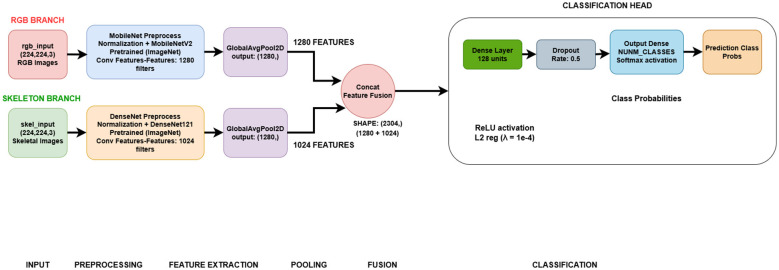
Workflow of the proposed methodology.

Accurate identification of medicinal plant species is important for pharmacognosy, agriculture, and biodiversity conservation. Prior image-driven classification methods suffer from high intra-class similarity, non-uniform illumination, and occlusion of plant leaves (Zhang et al., 2017; Atique et al., 2018; Pushpa and Rani, 2021). To bypass these limitations, a dual-branch deep learning framework, shown in Figure 5, that leverages both RGB leaf images and their corresponding skeletons was proposed, harnessing complementary spatial and structural leaf features. The proposed methodology was tested on a custom-made dataset consisting of 14,344 images, with each class represented by both RGB and its corresponding skeletonized leaves. RGB images have the texture, color, and shape cues needed to detect species-specific patterns. But in many medicinal plants, those leaves can be yellow, green, or brown, or exhibit a range of textures, depending on the environment, which can be confusing. Skeletonized images, which highlight the leaf's vein structure, offer a robust morphological abstraction invariant to lighting and color. These vein patterns are typically species-specific and biologically meaningful for classification. The proposed dual-branch structure enables parallel processing of these two data modalities using pretrained convolutional backbones (MobileNetV2 Sandler et al., 2018 for RGB, DenseNet121 Huang et al., 2017 for skeletons) for visual representation learning. By fusing high-level appearance and anatomical features, the dual-branch model improves robustness to morphological variation among similar species by capturing complementary cues not available from RGB or venation alone. This improvement reflects fine-grained discriminative stability rather than broader dataset-level generalization. This fusion is useful because it disambiguates intra-class similarities, improving the model's classification robustness. Besides architectural novelty, the methodology comprises strategies to handle class imbalance. A class-frequency-aware augmentation pipeline was used to strengthen data augmentation for minority classes, exposing them to stronger transformations and the majority classes to lighter ones, with class weighting during training. This ensured that the minority classes were learned sufficiently. This approach, along with transfer learning and regularization, offers a robust and scalable pipeline for medicinal plant classification. The proposed methodology was compared with models such as MobileNetV2 (Sandler et al., 2018), trained on RGB images only; DenseNet121 (Huang et al., 2017), trained on skeletonized images only; EfficientNetB0 (Tan and Le, 2019), trained on skeletonized images only; and DenseNet121, trained on fused images. The proposed methodology yielded better results than the mentioned models, indicating that it provides a reliable and robust medicinal plant classification pipeline.

### 3.4 Proposed flow

The workflow of the proposed methodology is shown in Figure 5. The workflow is divided into two parallel branches—the RGB branch and Skeleton branch. Each branch consists of coordinated preprocessing, feature extraction, feature pooling, and feature-level fusion of the extracted feature vector. The system takes RGB images and their corresponding skeletonized (leaf vein) images as inputs. The skeletonized images are obtained by applying various image processing techniques to the RGB images to extract the appropriate binary leaf-vein images. Color-mapping is applied to the skeletonized inputs to convert them from 1-channel to 3-channel images, and the resulting images are then fed to the model branch as input. Both branches include normalization and custom data augmentation using Albumentations (Buslaev et al., 2020), along with pipelines that adapt the frequency of each class to counter dataset imbalance. RGB inputs are processed by the MobileNetV2 (Sandler et al., 2018) model, pretrained on ImageNet with frozen weights, which extracts 1,280 high-level spatial features. Skeleton inputs are processed by DenseNet121 (Huang et al., 2017), which extracted 1,024 compact structural features. Each branch uses GlobalAvgPooling2D to condense the extracted spatial and structural features into robust feature vectors for fusion. After processing the inputs from each branch separately, the features from both branches are concatenated (1,024 + 1,280) to form a 2,304-dimensional joint representation, which is then passed to a shared classification head. The fused features are then processed by a dense layer (128 units), followed by dropout regularization, culminating in a softmax classifier that outputs the final class probabilities. Processing both the RGB and skeleton images separately before the fusion stage helps preserve the distinct strengths of the input data, maximizes the extraction of unique information, and prevents early-stage feature incompatibility from reducing the models' learning efficiency.

**Figure 6:**
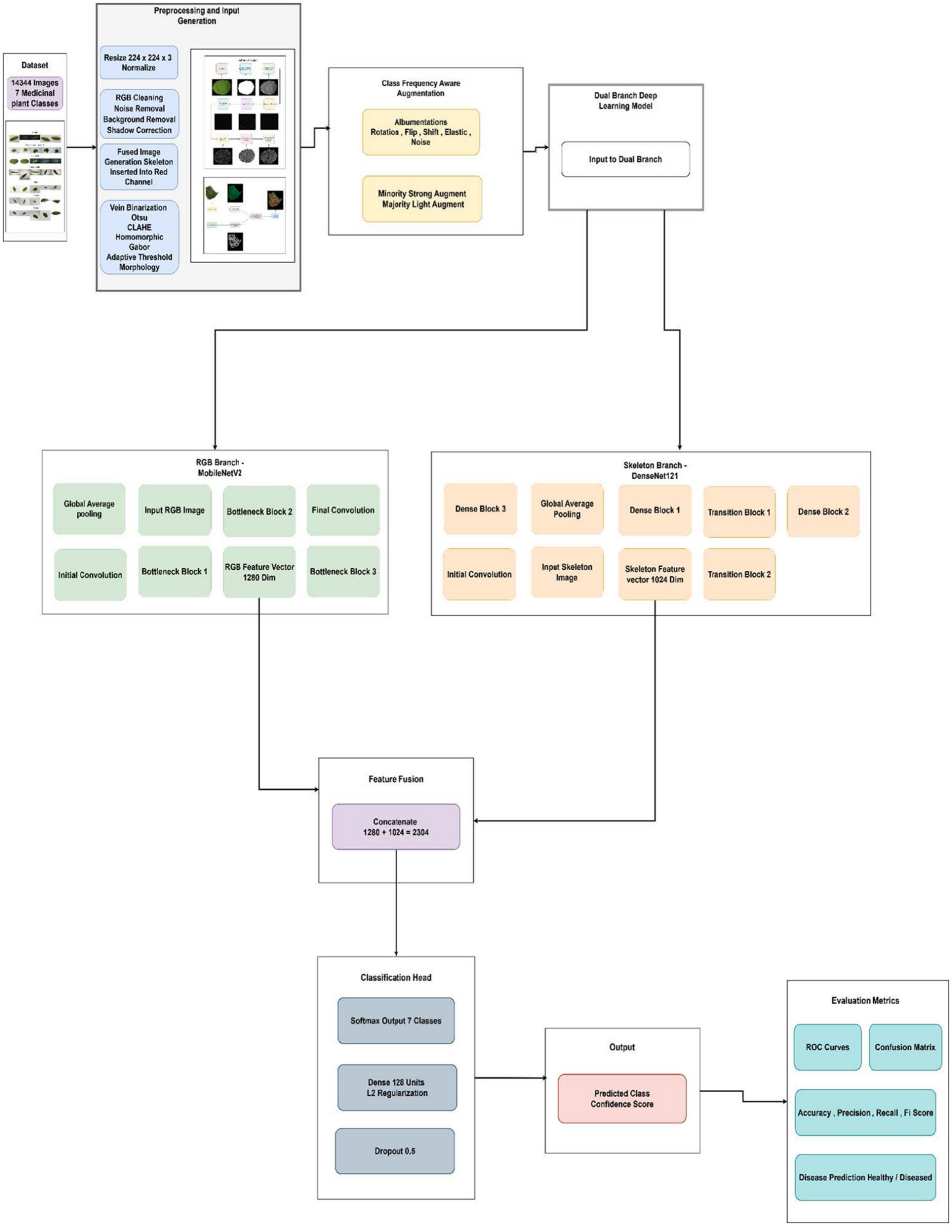
Architecture diagram of proposed methodology.

The architecture design of the proposed system leverages complementary information from two distinct data groups, RGB leaf images and their corresponding skeletonized leaf images. As illustrated in Figure 6, the system uses a dual-branch architecture, with each branch dedicated to extracting distinctive, biologically relevant features from its respective input type, before fusing them for robust classification.

The proposed model accepts two distinct inputs: RGB and Skeleton. RGB images of plant leaves are resized to a fixed spatial resolution of (224 × 224 × 3). These images preserve raw visual features such as texture, color, and shape. Skeleton input is binary images representing leaf venation, converted to 3-channel representations via color mapping to ensure compatibility with pre-trained CNN backbones. The skeleton input encodes topological and structural features of the leaf that are invariant to lighting, texture, and color. Both branches start with normalizing the input images, scaling the pixel values to match the pretrained model's expectation. This helps keep the training process stable and ensures compatibility with the pre-trained feature extractors. A customized data augmentation pipeline, built with the Albumentations library (Buslaev et al., 2020), was used to increase dataset diversity by applying random transformations such as rotations, flips, and shifts. To address class imbalance, the pipeline uses class-frequency-aware augmentation, applying stronger augmentations to the underrepresented class than to the majority classes. This ensures that the learning process is not dominated by the majority class. Exposing minority classes to stronger augmentation increases their contribution to the loss function. It enables the model to learn more stable and discriminative features from minority-class species, resulting in improved class-wise balance and more reliable predictions. This approach improves data representation and helps the model learn more about minority classes during training.

In the RGB branch, the normalized and augmented images are passed through a pretrained MobileNetV2 (Sandler et al., 2018) backbone, with its weights frozen to utilize its efficient depthwise separable convolutions while preserving the pre-trained knowledge. This branch outputs 1,280 high-level spatial and appearance-related features that capture patterns in color, texture, and global leaf geometry. In the skeleton branch, the skeletonized images are processed using a pretrained DenseNet121 (Huang et al., 2017) network. The dense connectivity of DenseNet121 enables feature reuse and facilitates the learning of fine-grained vein patterns. This branch outputs 1,024-dimensional feature vectors that capture critical information about leaf morphology and venation patterns. The feature extraction process for each branch ends with a 2D Global Average Pooling (GlobalAvgPool2D) layer, which reduces the spatial dimensions and produces compact feature vectors. This pooling operation reduces each feature map to a single scalar value, converting the high-dimensional convolutional outputs from MobileNetV2 (Sandler et al., 2018) (1,280 output features) and DenseNet121 (1,024 output features) into flat, compact feature vectors that capture global spatial and anatomical information, respectively.

The pooled feature vectors are concatenated to form a single 2,304-dimensional feature representation (1,280 from RGB + 1,024 from skeleton). This fusion is done at the feature level, ensuring that both visual and structural information is preserved and aligned before classification. The fused feature vector is passed to a shared four-layer classification head. The first layer is a dense layer comprising 128 units with ReLU activation and applies *L*2 regularization (λ = 1*e*−4) to reduce overfitting. This layer is followed by a dropout layer with a dropout rate of 0.5 that randomly deactivates half of the neurons during training. This further reduces the risk of overfitting by preventing co-adaptation among neurons. The output of this stage is then passed to a final dense layer whose output dimensionality equals the number of classes in the dataset. It uses the Softmax activation function to predict class probabilities. Finally, the class with the highest prediction probability is selected as the final predicted plant species.

#### 3.4.1 Design rationale and architectural choices

Based on the architecture, the choice of MobileNetV2 (Sandler et al., 2018) and DenseNet121 (Huang et al., 2017) as backbone networks was determined by their suitability for the application rather than solely by the number of parameters. Plant leaf classification is a very detailed classification problem in which there are often minor differences between classes that can be represented by the micro-textures, thickness of the leaf veins, how branches are formed, and relatively small morphological differences between the leaves.

Furthermore, although ultra-lightweight models such as ShuffleNet, SqueezeNet, MobileNetV3-Small, and GhostNet are efficient, they tend to have very shallow depth and limited capacity to express features; therefore, they are less suited to capturing the fine-grained characteristics of these structural/textural cues. This trade-off between model compactness and representational capacity has been well documented in the plant classification literature. Researchers (Pushpa and Rani, 2021) explicitly compared MobileNetV3-Large, DenseNet121, ResNet34/50, and VGG16 and found that the shallower, narrower variants have shown degraded performance on fine-grained medicinal leaf features.

On the other hand, MobileNetV2 (Sandler et al., 2018) and DenseNet121 provide a quality trade-off between efficiency and representational power, enabling robust extraction of both RGB appearance-based features and the fine-grained venation characteristics of the plants.

DenseNet121 was considered for the skeleton (venation) branch because of its dense connectivity, which enables feature reuse and improves gradient flow across layers. This allows the model to learn fine-grained structural details such as vein thickness, branching patterns, and morphological variations that are important for venation-based classification. Prior studies on leaf analysis and plant classification have reported the effectiveness of using DenseNet models in extracting complex structural and venation-related characteristics (Atique et al., 2018; Tang et al., 2023). This supports its suitability for skeleton representation in this work.

MobileNetV2 was considered for the RGB branch because of its depthwise separable convolution design, which efficiently captures spatial and texture-based features while maintaining computational efficiency. RGB leaf images primarily encode color, texture, and global shape cues, and lightweight models such as MobileNetV2 have demonstrated strong performance in appearance-based classification tasks.

Similarly, CNN attention-based variants were not selected. Attention-based modules can enhance feature refinement, but they increase parameters and memory usage costs. They are not suitable for this project's purpose of designing a deployable solution that is resource-efficient and suitable for deployment on mobile or field—based applications.

Additionally, dual-branch multimodal architectures may have their modality balances distorted by attention mechanisms, causing one branch to be overemphasized, thereby complicating the late-fusion strategy intentionally used to enable independent yet complementary learning of the plant's RGB and venation characteristics.

EfficientNetB0 was included in the comparison evaluation specifically as a skeleton-only baseline to assess whether its compound scaling strategy—which jointly optimizes the depth of the network, width, and input resolution-could yield stronger structural feature extraction from sparse vein images than the dense-connectivity approach of DenseNet121. Since the RGB branch was fixed to MobileNetV2 and the skeleton branch to DenseNet121 in our proposed dual-branch model, EfficientNetB0 was not evaluated on RGB or fused inputs, as doing so would yield an architectural configuration that differs from the controlled ablation of the skeleton branch backbone. Including it only as a skeleton-only competitor, thus enables a more controlled and direct comparison of 2 alternative CNN designs on identical skeletonized inputs, isolating the effect of the backbone choice for the vein morphology modality. As shown in Table 9, EfficientNetB0 achieved 94% accuracy on the skeleton images, whereas DenseNet121 achieved only 93% accuracy, a marginal difference that confirms the suitability of DenseNet121 as the skeleton-branch backbone and further validates the rationale for selecting the backbone in the proposed dual-branch architecture.

Moreover, due to their high data requirements and computational expense, both Vision Transformers (ViTs) and hybrid CNN–Transformer models were not utilized in this study. When used with smaller datasets (such as in terms of medicinal leaf datasets), which is a fine-grained classification task where the leaves exist with small vein and texture differences, ViTs are more likely to overfit and experience instability during training because they are not designed with local or translational invariant attributes, as are CNNs, thus necessitating pre-training on very large sets to demonstrate their best performance. Furthermore, the use of ViTs drastically increases inference time. It significantly increases memory usage compared to other non-transformer-based models, which runs counter to the goals of building a lightweight, deployable dual-branch model for use in an open-field setting. Furthermore, since the overall design of the model uses separate pathways for each modality, global attention-based token mixing would complicate the design of the late-fusion approach; therefore, ViTs and hybrid models were deliberately excluded to ensure performance and reliability for practical, deployable use.

### 3.5 Algorithm used

A total of five distinct algorithms were implemented for the classification of medicinal plant species using their RGB and skeletal image inputs: MobileNetV2 (Sandler et al., 2018) on RGB images, EfficientNetB0 (Tan and Le, 2019) on binary skeleton (leaf vein) images, DenseNet121 on skeleton images, DenseNet121 on fused images and the proposed dual-branch model that combines both RGB and skeleton features. Each model uses a convolutional neural network (CNN) architecture, either individually or in a dual-input fusion design.

#### 3.5.1 MobileNetV2 on RGB images

MobileNetV2 is a lightweight CNN that is designed for mobile and embedded vision applications. It utilizes depth-wise separable convolutions as formulated in Equation 9, and inverted residual blocks with linear bottlenecks to achieve efficiency. A depth-wise separable convolution factorizes the standard convolution into two operations:


$$\begin{array}{l} y=\alpha \left(W_{p}*\alpha \left(W_{d}*x\right)\right) \end{array}$$ (9)

where *W*_*d*_ is the depthwise convolution kernel applied per channel, *W*_*p*_ is the 1 x 1 pointwise convolution kernel, and α(·)is the non-linear activation (ReLU).

Given an RGB input *x*∈ℝ^224 × 224 × 3^, the MobileNetV2 backbone *f*_mb_(*x*; θ) outputs a feature tensor, which is subsequently globally pooled as shown in Equation 10.


$$\begin{array}{l} z=\text{GAP}\left(f_{\text{mb}}\left(x;\theta \right)\right) \end{array}$$ (10)

where GAP denotes the Global Average Pooling operation. The classification layer is a dense softmax layer applied over z as shown in Equation 11:


$$\begin{array}{l} ŷ=\text{softmax}\left(Wz+b\right) \end{array}$$ (11)

where *W* ∈ ℝ^*C*×*d*^ and *b* ∈ ℝ^*C*^, with *C* being the number of classes.

#### 3.5.2 EfficientNetB0 on skeleton images

EfficientNetB0 uses compound scaling to uniformly adjust network depth (D), width (W), and input resolution (R) as shown in Equation 12:


$$\begin{array}{l} D=\alpha \phi ,\;W=\beta \phi ,\;R=\gamma \phi \end{array}$$ (12)

where α·β^2^·γ^2^ ≈ 2 and α, β, γ ≥ 1. Here, α, β, and γ are constants, and ϕ is the scaling coefficient used to grow the model.

Given a binary skeleton image $x_{\text{bin}}\in {\mathbb{R}}^{224\times 224}$, a colormap transformation CM_jet_ is applied to transform it into an RGB form, as shown in Equation 13.


$$\begin{array}{l} x_{\text{rgb}}=\text{C}{\text{M}}_{\text{jet}}\left(x_{\text{bin}}\right)\in {\mathbb{R}}^{224\times 224\times 3} \end{array}$$ (13)

The EfficientNetB0 backbone feff, feature extraction, and pooling are shown in Equation 14:


$$\begin{array}{l} z=\text{GAP}\left(f_{\text{eff}}\left(x_{\text{rgb}};\theta_{\text{eff}}\right)\right) \end{array}$$ (14)

Classification is performed as shown in Equation 15:


$$\begin{array}{l} y=\text{softmax}\left(Wz+b\right) \end{array}$$ (15)

#### 3.5.3 DenseNet121 on skeleton images

DensetNet121 connects each layer to all the preceding layers to enhance the gradient flow and promote feature reuse, as shown in Equation 16. For the *l*th layer:


$$\begin{array}{l} x_{l}=H_{l}\left(\left[x_{0},x_{1},x_{2},\ldots ,x_{l-1}\right]\right) \end{array}$$ (16)

where [·] denotes channel-wise concatenation, and *H*_*l*_(·) is a composite function consisting of batch normalization, ReLU, and convolution.

Binary skeleton images xbin are first converted to RGB as shown in Equation 17:


$$\begin{array}{l} x_{\text{rgb}}=\text{C}{\text{M}}_{\text{jet}}\left(x_{\text{bin}}\right) \end{array}$$ (17)

The DenseNet121 backbone *f*_dn_ then outputs pooled features as shown in Equation 18:


$$\begin{array}{l} z=\text{GAP}\left(f_{\text{dn}}\left(x_{\text{rgb}};\theta_{\text{dn}}\right)\right) \end{array}$$ (18)

The classification head follows the pooling of the features as shown in Equation 19:


$$\begin{array}{l} y=\text{softmax}\left(Wz+b\right) \end{array}$$ (19)

#### 3.5.4 DenseNet121 on fused images

DenseNet121 was trained on fused images using the same backbone architecture described in Section 3.4.3, which was used to train the skeleton images. The fused input $x_{\text{fused}}\in {\mathbb{R}}^{224\times 224\times 3}$ was processed through the pre-trained DenseNet121 model, followed by Global Average Pooling. The pooled features are computed in the same manner as Equation 18 with *x*_fused_ as input.

The classification is computed as shown in Equation 20. The classification head consists of three main components. First, a dense layer with 128 neurons is applied using the ReLU activation function and an *L*2 regularization (λ = 10^−4^). This is followed by a dropout layer with a dropout probability of *p* = 0.5, and finally, the output is passed through a softmax layer to generate the predicted class probabilities, which are expressed as


$$\begin{array}{l} y=\text{softmax}\left(W_{o}\cdot \text{Dropout}\left(h\right)+b_{o}\right) \end{array}$$ (20)

Training uses the categorical cross-entropy loss with label smoothing (ϵ = 0.01) as shown in Equation 21:


$$\begin{array}{l} L=-\frac{1}{N}\overset{N}{\underset{i=1}{\sum }}\overset{C}{\underset{c=1}{\sum }}\left[y_{i,c}\left(1-\epsilon \right)+\frac{\epsilon }{C}\right]\log\left(y_{\text{pred},i,c}\right) \end{array}$$ (21)

where *N* is the batch size, *C* is the number of classes, *y*_*i, c*_ is the ground truth label for the *i*-th sample and *c*-th class, and *y*_pred, *i, c*_ is the predicted probability for the *i*-th sample and *c*-th class.

#### 3.5.5 Proposed dual-branch model

The proposed architecture fuses both the RGB and skeleton-based features through parallel CNN streams.

For RGB input *x*_rgb_ and skeleton input *x*_skel_, the RGB features and skeletal features are extracted as shown in Equations 22 and 23, respectively:


$$\begin{array}{l} z_{\text{rgb}}=\text{GAP}\left(f_{\text{mb}}\left(x_{\text{rgb}};\theta_{\text{mb}}\right)\right) \end{array}$$ (22)

$$\begin{array}{l} z_{\text{skel}}=\text{GAP}\left(f_{\text{dn}}\left(x_{\text{skel}};\theta_{\text{dn}}\right)\right) \end{array}$$ (23)

Feature fusion is performed by concatenation of the pooled features from the respective input branches, as shown in Equation 24:


$$\begin{array}{l} z_{\text{fused}}=\left[z_{\text{rgb}}+z_{\text{skel}}\right] \end{array}$$ (24)

The fused features pass through a fully connected layer with *L*2 regularization before final classification is done, as shown in Equation 25:


$$\begin{array}{l} y=\text{softmax}\left(Wz+b\right) \end{array}$$ (25)

### 3.6 Initialization and hyperparameters

The hyperparameters used for the proposed model are listed in Table 2, with the parameter name and value for each.

**Table 2:** Initialization and hyperparameters used for proposed model.

Parameter	Value
Image size	224 × 224
Batch size	16
Epochs	50 (with early stopping)
Optimizer	Adam
Learning rate	1 × 10^−5^ (Learning rate reduction on plateau)
Loss function	Categorical crossentropy (label smoothing = 0.01)
Class Weight balancing	Enabled
Pre-trained backbones	MobileNetV2 (RGB input), DenseNet121 (skeleton input)
Dropout rate	0.5
Dense layer units	128 (ReLU activation)
Regularization	L2 (1 × 10^−4^)

The proposed model was trained using the hyperparameters mentioned in Table 2. These were selected using an empirical, iterative tuning strategy informed by prior literature and preliminary validation experiments. Automated optimization techniques such as grid search or greedy algorithms were not used; the key hyperparameters, including learning rate, batch size, dropout rate, and regularization strength, were adjusted incrementally based on validation performance to ensure stable convergence and prevent overfitting.

### 3.7 Computational complexity and inference time

The computational efficiency of the proposed dual-branch framework is achieved through lightweight pre-trained CNN backbones and a late feature-level fusion approach. The RGB branch uses MobileNetV2, which employs depth-wise separable convolutions that significantly reduce the number of parameters and floating-point operations. The skeleton branch uses DenseNet121, which features dense connectivity that enables feature reuse and efficient gradient propagation without substantially increasing computational overhead. The convolutional layers of both models were initialized with ImageNet-pre-trained weights and kept frozen during training; as a result, training complexity is controlled by the shallow classification head, which consists of a single fully connected layer and dropout regularization. Feature fusion is implemented using simple concatenation followed by a dense layer, resulting in negligible additional computation.

The overall computational complexity of the proposed model can be expressed as the sum of the individual branch complexities, as shown in Equation 26:


$$\begin{array}{l} C_{\text{total}}=O\left(C_{\text{MobileNetV2}}+C_{\text{DenseNet121}}+C_{\text{fusion}}\right) \end{array}$$ (26)

where *C*_fusion_ corresponds only to feature concatenation and a small fully connected layer.

During inference, both branches run in parallel, resulting in near-linear scaling compared to single-branched models. The inference time of the proposed system can be approximated as shown in Equation 27:


$$\begin{array}{l} T_{\text{dual}}\approx T_{\text{RGB}}+T_{\text{skel}}+T_{\text{fusion}}, \end{array}$$ (27)

where *T*_RGB_ and *T*_skel_ denote the inference time of the MobileNetV2-based RGB branch and the DenseNet121-based skeleton branch, respectively, and *T*_fusion_ represents the lightweight overhead of feature concatenation and the classification head.

In practice, the relative inference time of the proposed model with respect to a single MobileNetV2-based model is expressed in Equation 28:


$$\begin{array}{l} \frac{T_{\text{dual}}}{T_{\text{MobileNetV2}}}\approx 1.6\text{–}1.8, \end{array}$$ (28)

This shows that the proposed model incurs only a modest increase in inference time, while remaining significantly more efficient than the ensemble and attention-based fusion approaches currently present. These characteristics make the proposed dual-branch framework suitable for real-world, resource-constrained deployment scenarios.

## 4 Results and discussion

### 4.1 Experimental setup

#### 4.1.1 Hardware and software requirements

All experiments for the proposed model were conducted on a remote high-performance GPU server accessed via a secure terminal connection. The training environment was equipped with two NVIDIA H100 GPUs connected via NVLink for high-bandwidth inter-GPU communication. The system operated in a headless configuration, with all commands executed via a terminal interface. GPU acceleration was enabled via CUDA 12.2 and cuDNN 8.9.

The baseline models were trained on a local machine equipped with an NVIDIA RTX 3050 GPU (6 GB VRAM), an Intel i5 CPU, and 16 GB of RAM, running Windows 11 with Python 3.10, TensorFlow 2.10, and the same supporting libraries used for the proposed model.

#### 4.1.2 Tools used for development

The proposed model was implemented in Python, with TensorFlow/Keras as the primary deep learning framework. Albumentations was used for data augmentation, and OpenCV for image processing. Evaluation metrics and visualizations were produced using Matplotlib and scikit-learn. The baseline models were implemented using the same framework to ensure consistency in training and evaluation pipelines.

#### 4.1.3 Thresholds and justifications

A class sample threshold of 800 was set to mitigate dataset imbalance. Classes that had fewer than 800 samples were classified as minority classes, and the remaining were classified as majority classes. Strong augmentation (elastic distortion, higher rotation angles, Gaussian noise) was used on the minority classes, whereas the majority classes were subjected to lighter augmentation to preserve the original data characteristics while introducing variability.

### 4.2 Detailed analysis of output plots and figures

#### 4.2.1 Proposed dual branch approach

The classification performance of the proposed model is shown in Table 3. The model achieved an overall accuracy of 97%, with a macro-averaged precision, recall, and F1-score of 0.96, 0.96, and 0.96, respectively.

**Table 3:** Classification report of proposed model.

Class	Precision	Recall	F1-score	Support
Arjun (new)	0.93	0.89	0.91	45
*Centella asiatica* (new)	0.99	1.00	1.00	104
Guava (new)	0.94	0.91	0.93	35
Malabar leaf (new)	0.97	1.00	0.98	89
Mint (new)	0.93	0.99	0.96	117
Neem (new)	0.99	1.00	1.00	141
Tulsi (new)	0.99	0.94	0.97	187

The results of the proposed model indicate an overall accuracy of 0.97, showcasing excellent performance across all classes. The model has shown near-perfect classification for the *Centella asiatica* and neem classes, both achieving a recall of 1.00, meaning it has correctly classified all instances of these classes without any false negatives. Other classes, such as Arjun and Guava, show slightly lower recall (0.89 and 0.91, respectively); their precision remains above 0.93, indicating that the model rarely misclassifies them as these species. The overall macro average (precision = 0.96, recall = 0.96, F1-score = 0.96) and weighted average (precision = 0.97, recall = 0.97, F1-score = 0.97) further confirm the model's consistent and balanced performance across all plant categories.

**Figure 7:**
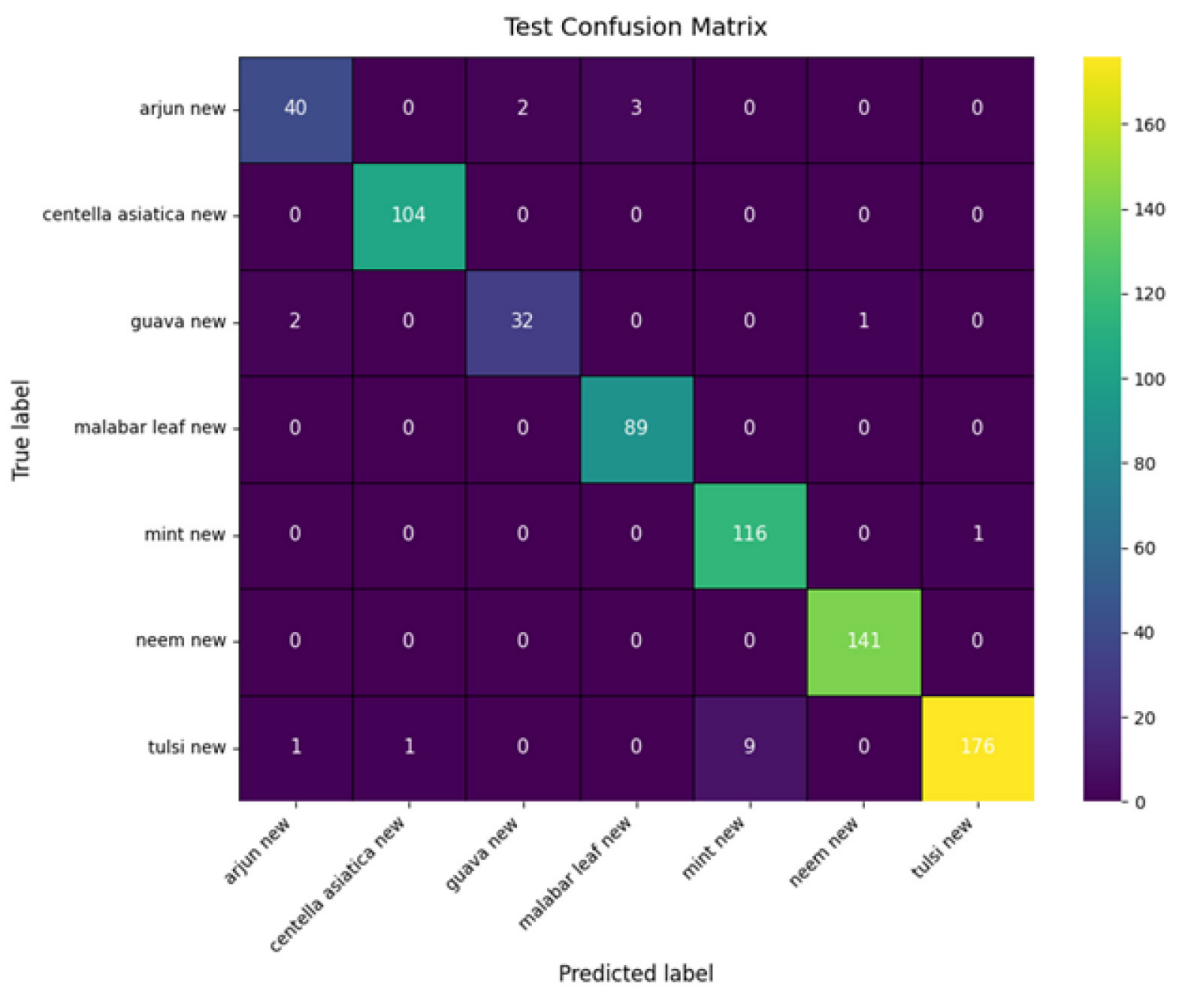
Confusion matrix of proposed model.

The confusion matrix illustrated in Figure 7 shows the distribution of the predictions across the seven classes for the proposed model. The majority of predictions are distributed along the diagonal, confirming that the model has classified them correctly. Perfect classifications are observed for the *Centella asiatica*, Mint, and Neem classes, and minor misclassifications are observed in species such as Arjun and Guava, likely due to overlap in color, shape, or patterns when viewed in RGB. However, integration of the skeleton vein pattern of leaves largely reduces such errors.

**Figure 8:**
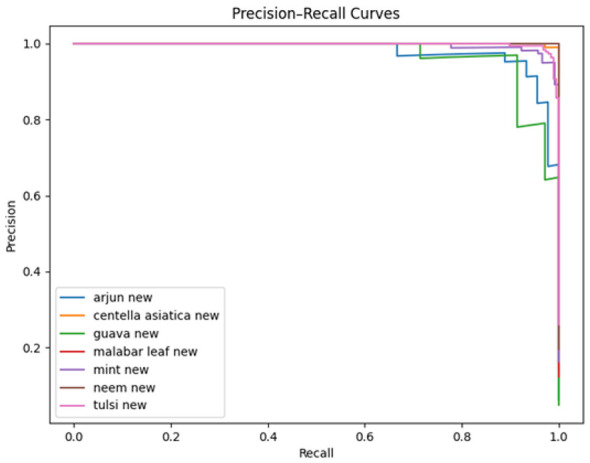
Precision-recall curve of proposed model.

The precision-recall curves illustrated in Figure 8 for the proposed model show that all classes achieve high precision across almost all recall ranges, with most maintaining precision above 0.95. The few dips in the value correspond to species with closely similar morphological characteristics (Arjun, Guava). The high area under the graph shows robustness even under class imbalance.

**Figure 9:**
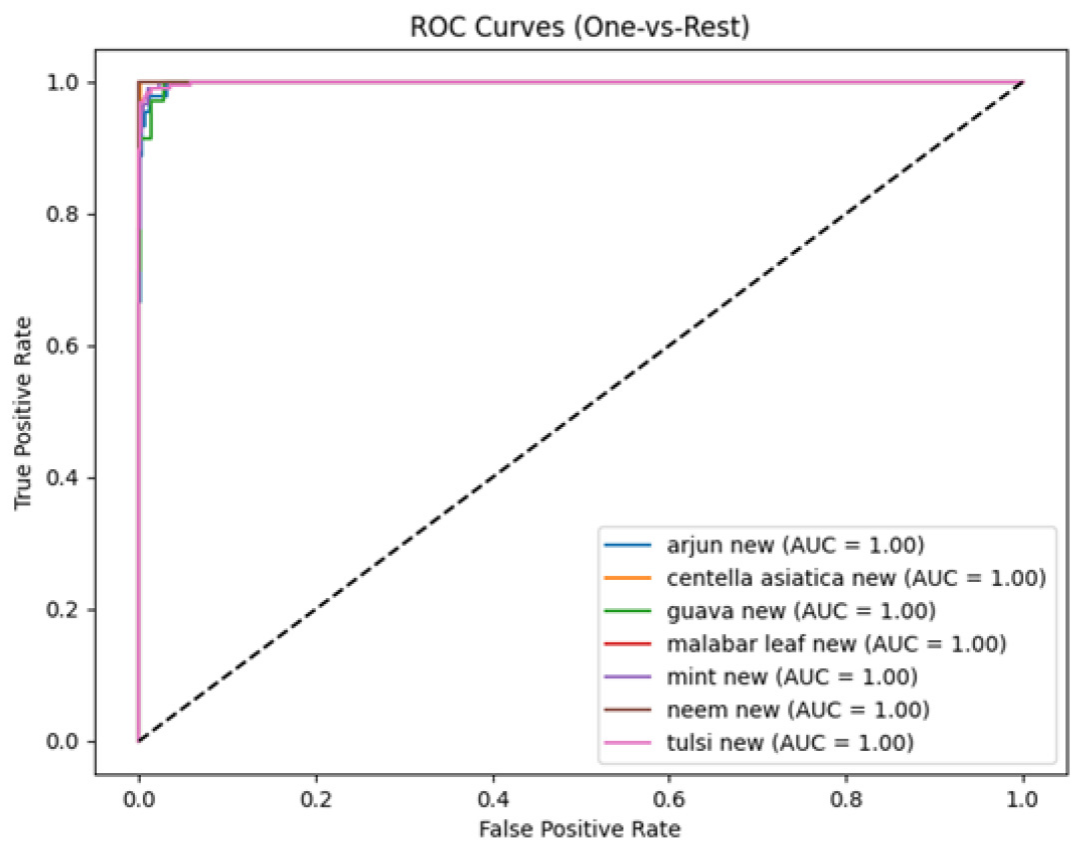
ROC curve of proposed model.

The ROC curves shown in Figure 9 indicate that all classes in the proposed model achieve an AUC of 1.00, indicating near-perfect separability in the feature space. The ROC curves remain close to the top-left corner of the graph, indicating the model's ability to classify plant species correctly and maintain low false-positive rates.

#### 4.2.2 DenseNet121 with fused images

The classification report of DenseNet121 with fused images is listed in Table 4. The classification Report of the fused image DenseNet121 model shows an accuracy score of 0.96 and macro-averaged precision, recall, and F1 score of 0.96, 0.95, and 0.96, respectively, while the weighted averages for precision, recall, and F1 score are all 0.96, showing consistent performance. *Centella asiatica* and Neem are perfectly classified, with perfect recall and a near-perfect F1 score of 0.99, reflecting their distinctive features. Guava and Malabar leaf achieve perfect recall and F1 scores of 0.93 and 0.99, despite smaller support, which showcases that even limited training data is feasible when shapes are distinctive. Mint has balanced performance but has some miss rate. Arjun exhibits high precision (0.93) but low recall and F1 scores of 0.87 and 0.90, respectively. The higher precision shows few false positives, but the lower recall indicates that some Arjun leaves are missed. Tulsi exhibited a high precision of 0.96 but a low recall of 0.91, which is lower than that of Neem or *Centella asiatica*, which reflects the model's tendency to under-recognize some Tulsi leaves.

**Table 4:** Classification report of fused image with DenseNet121 model.

Class	Precision	Recall	F1-score	Support
Arjun (new)	0.93	0.87	0.90	45
*Centella asiatica* (new)	0.98	1.00	0.99	104
Guava (new)	0.88	1.00	0.93	35
Malabar leaf (new)	0.99	1.00	0.99	89
Mint (new)	0.92	0.94	0.93	117
Neem (new)	0.99	1.00	0.99	141
Tulsi (new)	0.96	0.91	0.94	187

**Figure 10:**
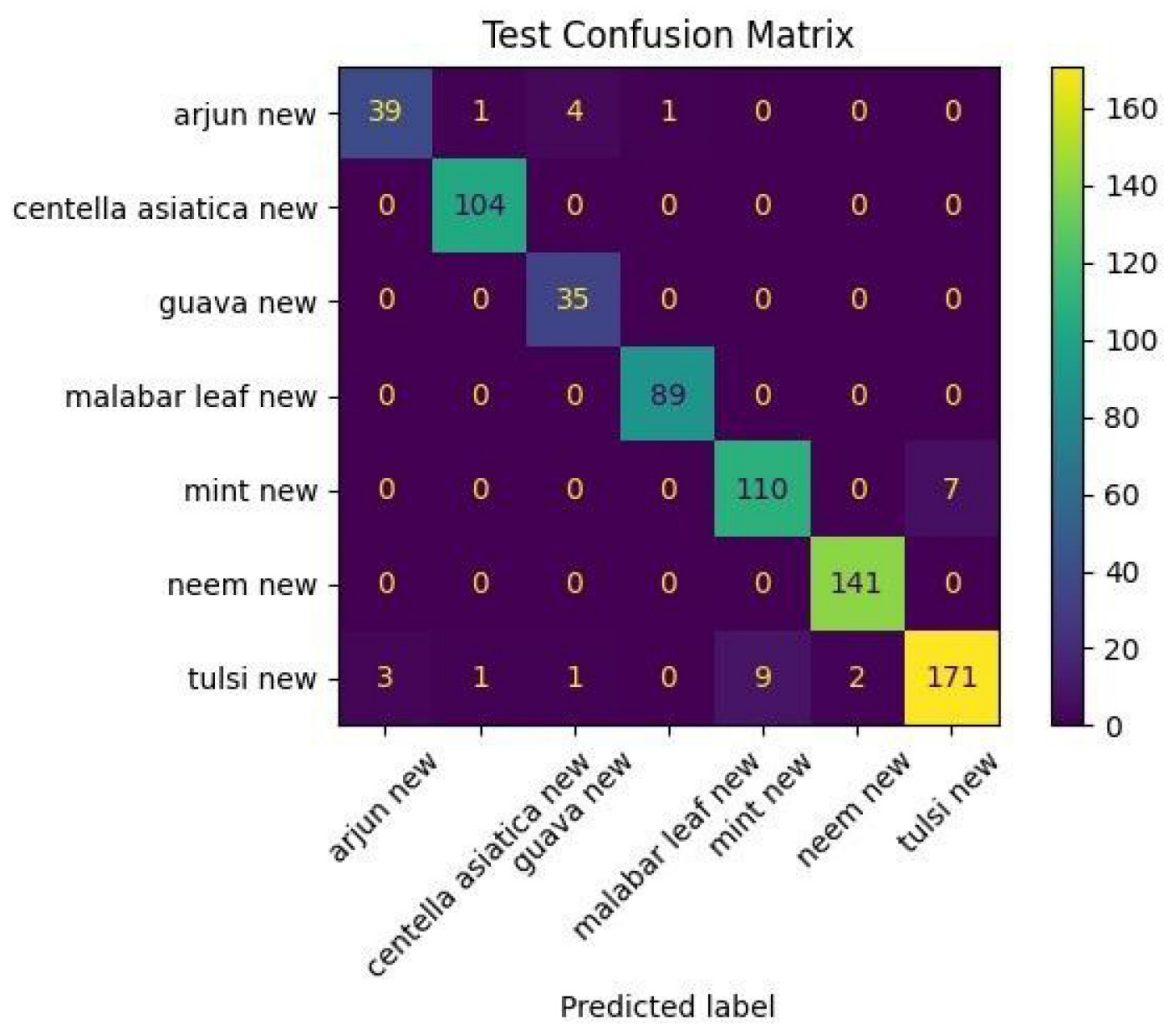
Confusion matrix of fused image DenseNet121 model.

The confusion matrix for DenseNet121 with a fused image model, illustrated in Figure 10, shows the model's test predictions across seven classes on 718 samples. Arjun has 39 correctly classified leaves with errors, with guava having the highest count, followed by Malabar leaf and *Centella asiatica*, indicating that certain Arjun leaf shapes resemble those of misclassified species. *Centella asiatica*, Guava, Malabar leaf, and Neem exhibit perfect accuracy with zero misclassifications, showing that their unique vein structures and leaf margins were well learned. Mint had 7 misclassified images classified as Tulsi, indicating significant morphological overlap between mint and Tulsi, such as similar serrated veins or venation, which the model struggles to distinguish. Tulsi had 16 misclassifications: 9 as mint, 3 as Arjun, 2 as neem, and 1 as guava. This error spread indicates that the Tulsi leaf appearance varies enough to occasionally overlap not only with mint but also with other unrelated species.

Due to the replacement of the red channel of the leaf image with the skeletonized vein images results in the DenseNet model a mix of real color information (green, blue) and the artificial vein lines (red), which results in breaking of the image's usual color/texture patterns and floods one channel with high contrast noise and scrambling of its feature statistics. Ultimately, this results in the vein outlines of different classes looking too similar, confusing the network, and leading to lower accuracy.

#### 4.2.3 DenseNet121 with skeletonized images

The classification report for DenseNet121 on skeletonized images is shown in Table 5. The classification report for the DenseNet121 model with the Skeletonized vein images shows an overall accuracy of 0.93, with macro-averaged precision, recall, and F1-score of 0.91, while the weighted averages for precision, recall, and F1-score are all 0.93, indicating consistent performance. Arjun has displayed a precision of 0.88 and a recall of 0.71, which shows that many Arjun specimens were misclassified. *Centella asiatica* achieved classification metrics such as a precision of 0.97, a recall of 0.94, and an F1-score of 0.96, indicating near-perfect classification. The same applies to Malabar leaves, with classification metrics exceeding 0.95. Mint achieved a precision of 0.86, which is moderate, but displayed a very good recall of 0.97 and a decent F1-score of 0.91, indicating a good classification performance. Neem has achieved the best classification metrics, with a precision, recall, and F1-score of 0.99, showcasing near-perfect performance. Tulsi exhibits a good precision score of 0.97, a relatively low recall of 0.89, and an F1-score of 0.93, indicating that some significant misclassifications might have occurred for that class.

**Table 5:** Classification report of DenseNet121 on skeletonized images.

Class	Precision	Recall	F1-score	Support
Arjun (new)	0.86	0.71	0.78	45
*Centella asiatica* (new)	0.97	0.94	0.96	104
Guava (new)	0.78	0.89	0.83	35
Malabar leaf (new)	0.95	0.98	0.96	89
Mint (new)	0.86	0.97	0.91	117
Neem (new)	0.99	0.99	0.99	141
Tulsi (new)	0.97	0.89	0.93	187

**Figure 11:**
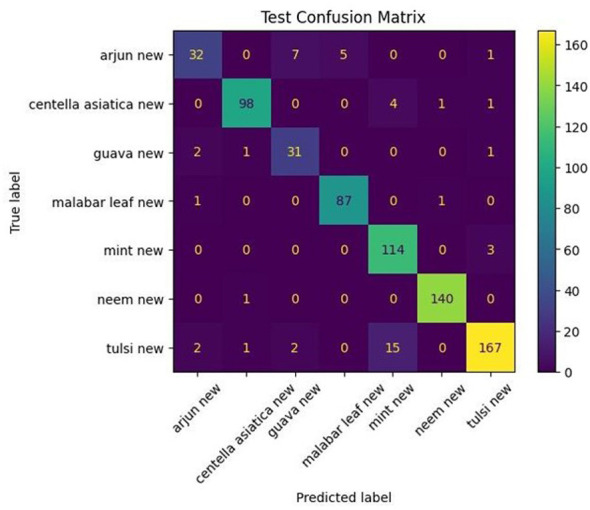
Confusion matrix of DenseNet121 on Skeletonized images.

The confusion matrix of the DenseNet121 Skeletonized vein model, illustrated in Figure 11, shows that Arjun has many misclassifications, and Guava is among the most misclassified, suggesting similar vein structures and outlines to Guava. Furthermore, *Centella asiatica* was occasionally confused with Tulsi and Neem, indicating similar vein structures. Guava also exhibited some misclassifications with Arjun and other leaves. While the Malabar leaf was classified correctly almost every time, with only two misclassifications, which showcased high discriminability of vein features and few overlaps at decision boundaries. Mint also achieved a near-perfect accuracy with only 3 misclassifications, with Tulsi indicating similar vein structures and outlines, which were already observed with other models, which displayed similar misclassifications. Neem achieved the best classification, with only a single misclassification, indicating perfect separation from the other classes. Tulsi, on the other hand, had the most misclassifications, with the majority classified as mint, due to similar vein structures and high intra-class variability. Similar misclassifications were observed in other models.

The DenseNet121 model excels by reusing rich feature patterns across layers in full-color, texture-rich images. However, when given the skeletonized vein image, there is too little unique information, as the veins are just single thin lines without any surface detail and have only a single channel, resulting in low information to exploit their dense connectivity. As many classes share similar vein patterns, when they are reduced to skeletonized vein images, the model is not able to distinguish them well, resulting in mixed feature maps and lower classification accuracy.

#### 4.2.4 EfficientNetB0 with skeletonized images

**Figure 12:**
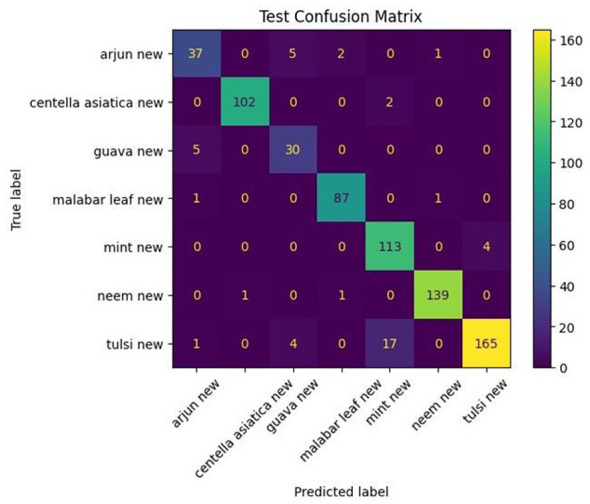
Confusion matrix of EfficientNetB0 on skeletonized images.

The classification report of EfficientNetB0 with skeletonized images is listed in Table 6. The classification report for the EfficientNetB0 model on skeletonized vein images shows an overall accuracy of 0.94, demonstrating strong generalization capability. The macro precision of 0.91, macro recall, and macro F1-score of 0.92, and the weighted averages for precision, recall, and F1-score are all 0.94, indicating improved performance compared to the previous model and balanced, consistent performance across all classes. *Centella asiatica*, Malabar leaf, and Neem achieved near-excellent performance, with precision, recall, and F1 scores above 0.97, while Guava exhibited the lowest precision, indicating false positives in which other leaves are classified as Guava. Arjun exhibited low precision, along with the lowest recall and F1 Scores, indicating continued difficulty with this class. Mint exhibited a good recall of 0.97 but lower precision and F1-score, suggesting some non-mint leaves were classified as mint. Tulsi exhibited good precision and F1-score but a low recall of 0.88, indicating that some Tulsi leaves were misclassified. The confusion matrix results for the EfficientNetB0 with the skeletal image model, illustrated in Figure 12, show that *Centella asiatica*, Malabar leaf, and Neem achieved near-perfect classification with only two misclassified images each, while Arjun had seven misclassified images. The most commonly misclassified items were Guava, similar to the other DenseNet121 model (skeletonized images), indicating similar leaf recognition pattern variations. The same applies to Guava, with five images misclassified as Arjun. Mint exhibited similar confusion with Tulsi, with four images being misclassified as Tulsi. But this confusion is lower than that of the DenseNet121 model. The same also applies to Tulsi, having the majority of misclassified images, as Tulsi indicates persistent feature overlap between these classes.

**Table 6:** Classification report of EfficientNetB0 on skeletonized images.

Class	Precision	Recall	F1-score	Support
Arjun (new)	0.84	0.82	0.83	45
*Centella asiatica* (new)	0.99	0.98	0.99	104
Guava (new)	0.77	0.86	0.81	35
Malabar leaf (new)	0.97	0.98	0.97	89
Mint (new)	0.86	0.97	0.91	117
Neem (new)	0.99	0.99	0.99	141
Tulsi (new)	0.98	0.88	0.93	187

The lower accuracy is due to the model's ability to extract rich color, texture, and edge details from multi-channel images. But when fed only single-channel vein skeletons, the sparse lines lack color or texture features, and the model has too little information to learn distinct features. Because the vein patterns are similar across many classes, the model cannot form clear class boundaries, leading to overlapping representations and lower accuracy.

#### 4.2.5 MobileNetV2 with RGB images

**Figure 13:**
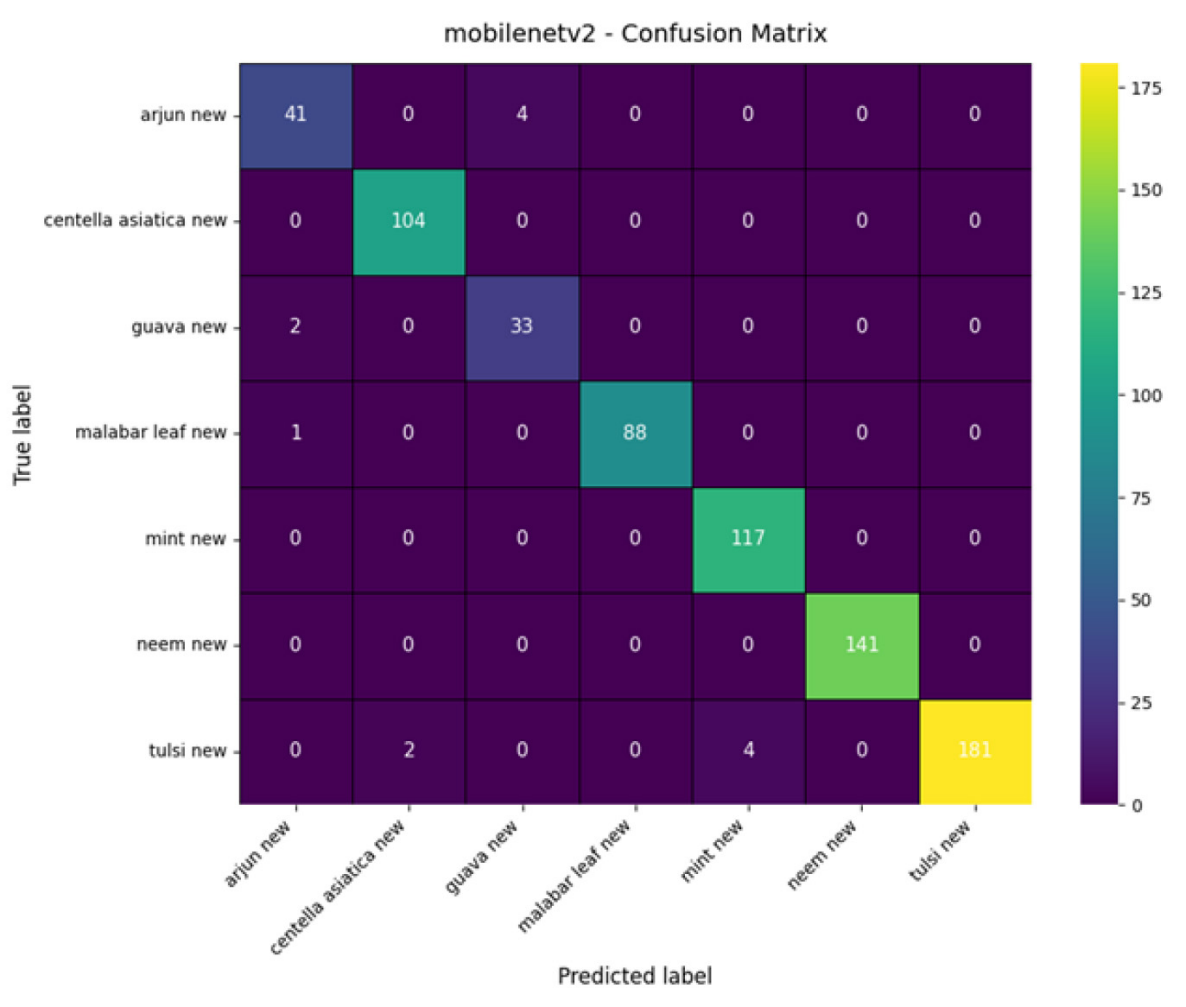
Confusion matrix for MobileNetV2 with RGB images.

The classification report for MobileNetV2 on RGB images is shown in Table 7. The MobileNetV2 model trained on RGB images alone achieved an overall test accuracy of 0.98 (Table 9), showcasing excellent classification performance. The macro-averaged precision, recall, and F1-score are all 0.97, while the weighted averages for precision, recall, and F1-score are all 0.98, indicating high consistency across all classes. The model's per-class F1-scores range from 0.92 to 1.00, with both the macro and weighted averages in the 0.97-0.98 range. The confusion matrix illustrated in Figure 13 shows that misclassifications were fewer and primarily occurred between visually similar species, such as Arjun and Guava, or Tulsi and Mint.

**Table 7:** Classification report of MobileNetV2 with RGB images.

Class	Precision	Recall	F1-score	Support
Arjun (new)	0.93	0.91	0.92	45
*Centella asiatica* (new)	0.98	1.00	0.99	104
Guava (new)	0.89	0.94	0.92	35
Malabar leaf (new)	1.00	0.99	0.99	89
Mint (new)	0.97	1.00	0.98	117
Neem (new)	1.00	1.00	1.00	141
Tulsi (new)	1.00	0.97	0.98	187

Although MobileNetV2 trained on RGB alone achieved a slightly higher overall accuracy, this is likely due to the model being pretrained using ImageNet, which provides extensive exposure to diverse natural textures, shapes, and plant-like patterns.

Due to pre-training, the model's ability to classify plant species using RGB visual cues is enhanced. Conversely, the proposed model leverages complementary structural information from skeletonized plant leaf vein images, capturing specific vein patterns that are robust to variations in lighting, background, and leaf coloration. Fusing RGB and skeletal information yields only a minimal overall difference in accuracy on the current test set (7 out of 718). This small difference is not statistically meaningful, and the dual-branch model compensates for it by encoding illumination-invariant venation features alongside RGB information. Because venation patterns are known to be robust to various lighting and color conditions (Atique et al., 2018), the proposed model is expected to be more robust in real-world settings where appearance-based cues are unreliable.

### 4.3 Comparative performance analysis with contemporary works

Accurate classification of medicinal plant species remains a challenging problem due to high intra-class similarity, inter-class variations caused by various environmental factors, and morphological variabilities in leaves. Prior works on the classification of these plants rely solely on RGB imagery that has shown its limitations in achieving robust results under non-uniform lighting and occlusions (Zhang et al., 2017; Pushpa and Rani, 2021; Atique et al., 2018). The proposed dual-branch framework achieves competitive performance compared to prior work by incorporating skeletonized leaf-vein images alongside RGB images of plant leaves. This structural information is invariant to color and lighting changes, allowing the model to correctly classify leaves that might otherwise have been misclassified. The 97% overall accuracy achieved by the model shows an improvement over prior benchmarks, validating the advantage of multimodal feature fusion for medicinal plant recognition. The performance comparison of existing models and the proposed model is listed in Table 8.

**Table 8:** Performance comparison between existing and proposed model.

Study (year)	Modality	Model/dataset (domain)	Metric reported	Performance	Notes
Tan and Chang (2019)	CNN (D-Leaf); vein morphometrics + TL AlexNet	Lab-controlled leaf dataset	Acc.	94.88%	Sobel-segmented venation + classical ML (SVM/ANN/KNN/NB/CNN) AlexNet (60M) parameters.
Rana et al. (2021)	Lightweight DCNN (AyurPlantNet)	Ayurvedic medicinal plant species (Indian)	Acc.	92.27%	Compared vs. MobileNetV3-Large, DenseNet121, ResNet34/50, VGG16.
Multi-modal Fusion Study (2021)	Ensemble (MobileNetV2 + InceptionV3 + ResNet50; weighted avg.)	Custom medicinal leaf dataset, 30 classes, clean lab-captured images	Acc. (CV)	99.66%	3- and 5-fold CV; transfer-learning features + Softmax head Total Combined 48.8M+ parameters and only 1,547 training images.
Sarma et al. (2020)	Dual-branch (1D-CNN hyperspectral + MobileNetV3 RGB)	Maize varieties	Acc. (5-fold)	98.75%	Lightweight (2.53M params); gated fusion + CBAM attention.
Singh D. et al. (2020)	Dual-branch (CNN local + token/ViT global; attention fusion)	KLH herbs (multi-organ, in-the-wild) Plant Village Dataset	Top-1	94.7%	21.6M params; outperforms ResNet-152 (89.2%) & ViT-B (91.4%).
Murugavalli and Gopi (2025)	Vision Transformer (PLA-ViT)	ViT-based leaf disease detection (agricultural leaves), Plant Village Dataset	Acc.	98.7%	Improved robustness to illumination and background variation (Murugavalli and Gopi, 2025).
Aboelenin et al. (2025)	Hybrid CNN–ViT (local CNN + global transformer)	Medicinal herb leaves (disease classification), PlantVillage—apple and corn disease only	Acc.	98.0%	Joint extraction of texture and structural cues; stronger fine-grained discrimination (Aboelenin et al., 2025).
Jia et al. (2025)	Hybrid CNN–Transformer (ConvTransNet-S)	PlantVillage + in-field crop disease	Acc.	98.85% (Plant Village), 88.53% (in-field crop disease)	Lightweight hybrid; optimized for mobile/edge devices with balanced performance (Jia et al., 2025).
(Karaoǧlan and Karadeniz 2024)	Vision Transformer (pure ViT classifier)	Leaf morphology dataset (clean + natural images)	Top-1 Acc.	95.2%	Token-based modeling for fine morphological cues shows a trend toward transformer-driven plant ID (Karaoǧlan and Karadeniz, 2024).
Proposed work	Dual-branch RGB (MobileNetV2) + skeleton (DenseNet121); late fusion	Multi-source medicinal leaves (custom, paired RGB-skeleton)	Acc.	97.0%	Class-frequency-aware augmentation; stronger on morphologically similar pairs 3.4M + 8.06M parameters.

The performance of the proposed dual-branch medicinal plant classification model was compared with contemporary studies employing RGB-based, venation-based, and multimodal approaches, as shown in Table 8. RGB-only models, such as Ayur-PlantNet (Pushpa and Rani, 2021), achieved up to 92.27% accuracy but remain sensitive to illumination and background variations. Venation-based methods, like DenseNet-169 on Canny-extracted venation features (Atique et al., 2018) and the D-Leaf CNN (Tan and Chang, 2019), reported accuracies of 95.72% and 94.88%, respectively, yet may overlook key color and texture cues. Ensemble and advanced fusion models, such as EDL-AMLI (Sachar et al., 2021) and MPR-Net (Tang et al., 2023), achieved higher accuracies of 99.66% and above but at the cost of increased complexity and computational demands. Conversely, the proposed model offers a balanced solution, combining RGB and skeletonized venation features in a lightweight dual-branch architecture to deliver competitive accuracy while maintaining efficiency and real-world applicability. Notably, direct accuracy comparisons across studies in Table 8 are inherently limited by differences in dataset difficulty, imaging conditions, and task domain, and should therefore be interpreted with appropriate caution.

### 4.4 Comparison of various approaches

The performance comparison among the different models is presented in Table 9, showing accuracy, precision, recall, and F1-score for each model.

**Table 9:** Model performance comparison.

Model	Input type	Accuracy (%)	Macro precision	Macro recall	Macro F1-score
MobileNetV2	RGB only	98.0	0.97	0.97	0.97
EfficientNetB0	Skeleton only	94.0	0.91	0.92	0.92
DenseNet121	Skeleton only	93.0	0.91	0.91	0.91
DenseNet121	Fused image	96.0	0.95	0.96	0.95
Proposed work	RGB + skeleton	97.0	0.96	0.96	0.96

From Table 9, the MobileNetV2 trained on RGB images achieved the highest overall accuracy (98%) and better macro-averaged metrics than the proposed dual-branch model. Although the MobileNetV2 model performs slightly better than the proposed approach in this experimental setting, the proposed dual-branch model provides a more comprehensive feature representation by integrating the spatial RGB appearance features alongside illumination-invariant venation features of leaves. This allows the capture of complementary appearance and venation characteristics within a unified framework.

### 4.5 Improvements and enhancements

The improvements observed in the results are achieved through design choices such as using a dual-branch fusion approach for RGB and Skeleton features, which provide complementary structural and spatial information, enabling the model to classify plant species under various environmental conditions and constraints. Use of Class-frequency-aware augmentation helped improve the representation of minority classes, enabling the model to learn all classes more evenly and leading to balanced recall and precision during classification. The use of pre-trained model backbones, such as MobileNetV2 and DenseNet121, helped leverage transfer learning for more robust feature extraction while keeping computational costs manageable.

The use of regularization techniques such as dropout and an L2 penalty improved the model's performance on unseen data and reduced overfitting despite the model's high capacity. The combination of these factors improves the proposed model over single-branch baseline models in medicinal plant classification.

## 5 Discussion

Through experimental findings, the authors have shown that integrating RGB and Skeletonized Venation Images within a Dual-Branch Deep Learning Framework yields competitive classification performance by leveraging complementary structural information. Although traditional RGB methods do provide rich texture, shape, and color information, their performance can vary depending on environmental factors such as illumination, shadows, and background noise, whereas venation-based methods provide a morphological basis that is relatively stable under varying conditions, including lighting. However, they do not provide any surface-level appearance details that are important when distinguishing visually similar species.

Based on the authors' findings, using a single modality alone does not provide sufficient strength for identifying plant species in real-world situations. The dual-branch fusion framework leverages the strengths of both modalities to provide a more effective means of identifying plant species. RGB can capture overall shape and texture information, while Skeleton can capture localized, species-specific venation patterns. This has been especially useful when a particular plant species exhibits high intra-class variation or morphological overlap.

The remarkable increase in Mint and Tulsi classification confirms the merit of multimodal fusion. Because of the similarity in the shape and texture of leaves, it is difficult to differentiate between the two species using RGB alone; however, the venation pattern for each species differs subtly yet consistently, so the skeleton branch provides the discriminatory detail that RGB models would miss.

Other classes, such as Neem and *Centella asiatica*, which also have unique venation patterns, benefited from the venation modality, thereby increasing the reliability of the predictions. The fact that the model achieved a 97% accuracy rating demonstrates the success of (a) class-frequency-aware augmentation which reduced bias toward the class with the highest number of examples; (b) transfer learning which enabled the model to learn from a small amount of data; and (c) feature-level fusion that bypassed interference at the beginning stages of the processing of the modalities.

Finally, the lightweight and scalable nature of the proposed model makes it suitable for field or mobile-based applications such as the authentication of herbal drugs, the monitoring of ecosystems, and the conservation of biodiversity. Future research will likely focus on extending the multimodal fusion model framework to include additional plant organs (e.g., flowers, stems), multi-spectral imaging, and transformer-based fusion models.

## 6 Conclusion

This study presented a dual-branch deep learning framework for medicinal plant species classification that uses both RGB images and skeletonized leaf-vein pattern images. The architecture employs two input-specific pre-trained CNN backbones—MobileNetV2 for RGB images and DenseNet121 for skeletonized inputs—followed by late feature-level fusion. This architecture enables the proposed system to preserve and leverage the unique strengths of each input data source, such as texture, color, and shape cues from RGB images, and illumination-invariant morphological structures from skeletonized images. The proposed system is trained and evaluated on a custom dataset of 14,344 RGB-skeleton pairs. The proposed model achieved an overall accuracy of 97%, with a macro-averaged F1-score of 0.96 and a weighted F1-score of 0.97. Several classes, like neem and *Centella asiatica*, achieved perfect recall and near-perfect precision. Even for visually challenging plant species such as Arjun and Guava, the proposed model achieved strong F1-Scores of 0.91 and 0.93, respectively. Although the accuracy of the dual-branch model is slightly lower than the RGB-only MobileNetV2 baseline, we believe it uses both RGB and venation as complementary cues. It will provide greater overall class-wise stability for visually similar morphological species under real-world conditions, particularly when image quality varies.

The proposed deep learning models for classifying medicinal leaves are encouraging, with multiple aspects requiring improvement and offering avenues for future studies to enhance the system's accuracy, efficiency, and reliability in real-world settings. A notable area of growing popularity is the use of Vision Transformers for image classification, and these architectures have shown promising results that exceed those of their CNN counterparts. The integration of such Vision Transformers into the proposed methodology will greatly increase classification accuracy for leaves and improve real-world classification. Exploration of improved fusion strategies, such as attention-based fusion and cross-attention, will help achieve better leaf and vein fusion, and other features, such as texture and shape, can also be integrated to further optimize the model and improve its classification performance. Integrating explainable AI (XAI) techniques into the methodology will clarify which features or regions of a leaf are most crucial for predictions, providing valuable insights for botanists and researchers. Furthermore, adding more data is very useful for improving model performance.

Future work will explore lightweight CNNs, attention-based enhancements, and Vision Transformer or hybrid CNN–Transformer architectures as larger datasets and additional computational resources become available, to evaluate their potential to further improve efficiency and fine-grained classification performance.

## Data Availability

The datasets used in this study were compiled from publicly available sources, including Mendeley Data and open-access plant image repositories. All raw datasets used for compilation are accessible through their respective public links cited in the References. The processed dataset generated for this study (paired RGB and skeletonized images) is available from the authors upon reasonable request.
